# Clinical Review and Hospital Reports

**Published:** 1835-10-01

**Authors:** 


### Clinical Review.

### Pennsylvania Hospital.

Report of Cases Treated in the Surgical Wards. By T. S. Kirkbride, M. D. Resident Physician.*

This Report contains some very interesting cases, and is worthy the attention of those who love to study facts, the only genuine philosophy. We shall endeavour to abridge the details of the cases as far as is consistent with the preservation of their intrinsic interest and value. Two instances of strangulated hernia are recorded, each displaying some peculiar features.

### I. Hernia.

Case 1. *Strangulated Congenital Hernia—radical cure from placing the Testicle at the Neck of the Sac.*

J. B. æt. 22, a hack-driver, was admitted Aug. 30, 1833, at half-past 11 A.M. with strangulated scrotal hernia. The symptoms of strangulation had existed since early the preceding evening. Ineffectual attempts at reduction had been made.

On admission, the scrotal swelling large and tense, and the parts very tender; he suffers severe pain; has a quick and feeble pulse, cold skin, and great anxiety of countenance. He vomits occasionally, and has hiccup; after trying the usual means for restoring the intestine, an operation was performed by Dr. Barton at 1 P. M. Upon opening the sac, it was found filled with a dark fluid almost like blood, and having a peculiarly unpleasant odour, strongly resembling that of gangrene; the intestine also presented a very dark appearance. Some adhesions existed between the tunica vaginalis and the intestine; the point of stricture was at the neck of the sac, and so close, that a director was introduced with some difficulty. After returning the intestine into the abdominal cavity, it was evident that so large an opening existed, that if treated in the usual way, the patient would be liable to returns of the accident, or at all events, would be always compelled to wear a truss. But the testicle had never descended into the scrotum, being retained in the groin by the shortness of the cord, where it lay in contact with the intestine; it was therefore introduced into the mouth of the sac, with the prospect of adhesions taking place so as to prevent the possibility of another protrusion, and thus to effect a radical cure. The parts were neatly adjusted over it with sutures and compresses, secured by the T-bandage.

The patient was ordered fifty drops of laudanum, and small quantities of barley water. On the next day there was slight pain in the stomach, and a disposition to nausea. The bowels were not relieved until after two doses of castor oil had been taken. On the 2nd September there was slight erysipelatous inflammation in the groin—the abdomen was tympanitic—there was vomiting. Fomentations and mutton broth were ordered. Relief occurred in the evening, and next day there was a slight discharge of purulent matter from the wound. After this nothing of consequence took place. The wound united, in part by adhesions and in part by granulations, and in October, the patient would seem to have been dismissed from the hospital cured. In June, 1834, he met Dr. Kirkbride, and informed him that since he left the institution, he had been following his usual occupation in his usual way. For a few weeks he occasionally had pains shooting through the abdomen. Subsequently he had suffered no inconvenience, and had required no truss in consequence of the firmness of the parts and the perfect closure of the opening.

Dr. Kirkbride offers the following observation:—

* American Journal of the Medical Sciences, February, 1835.

"In this case the point of particular interest is the accomplishment of a *radical cure,* effected by the novel mode of filling up the mouth of the sac by placing the testicle in it, and by its subsequent adhesions to the neighbouring parts, without, in all probability, any interference with the functions of the gland."

There is one practical point to which we would direct a moment's attention. It will be observed that on the third day after the operation, the patient was attacked with irritability of the stomach, vomiting, and a tympanitic condition of the abdomen, and there also appeared an erysipelatous blush about the groin. Mutton broth and fomentations removed the symptoms. We have on several occasions witnessed symptoms of this character, but of greater severity, occurring after the operation for hernia. On the second or third day, the patient has presented a frequent pulse, a hot skin, a foul tongue, a tympanitic state of the abdomen attended with tenderness on pressure, and general depression, if not prostration, and these symptoms have manifestly depended on the formation of some matter in or about the wound. We have seen a patient leeched and bled and calomelized under these circumstances, when the result has proved that fomentations and poultices to the part, and perhaps such nutriment, as broth or arrow-root, would have been more appropriate measures. It is very desirable to avoid peritoneal inflammation, yet many individuals require judicious support, and not depletion, after the operation for strangulated hernia. This is frequently the case with elderly persons.

CASE 2. *Strangulated Femoral Hernia—Sloughing of the Sac—Recovery.* Susan Baker, æt. 50, admitted Aug. 23, 1834, at noon, with strangulated femoral hernia. The hernia had existed for two years, but was always reduced in the recumbent posture, until four days prior to her admission, since which it could not be returned. She had had no motion from the bowels for five days. Soon after the descent of the tumor she began to suffer from pain in the abdomen. Vomiting succeeded; and for several hours previous to her being brought into the hospital the matters vomited were stercoraceous. The skin was cool and moist, the pulse frequent and feeble, the countenance anxious. The operation was immediately performed by Dr. Barton.

The sac was found to be of an unusual thickness, and contained but a small portion of fluid; the intestine had rather a dark colour, but did not appear to be gangrenous. So close was the stricture, that a director was passed with difficulty. After enlarging the opening slightly, the intestine was returned into the abdomen with considerable facility. The patient bore the operation well, and her pulse, immediately after, was better than at the time of her admission.

*Half-past* 4 *p. m.* The patient has vomited several times since the operation; the first discharge was of a yellow colour, and offensive, but the last two or three appear to be bile, mixed with the secretions from the stomach. She has pain in the abdomen just before vomiting, but not at any other time. Her pulse is 100, soft, and regular; her skin is warm and dry; tongue slightly furred posteriorly, and inclined to dryness. ℞. Acid. tartar, ʒij.; Aquæ, ℥vj.; ft. sol. ℞. Sup-carb. sod. ʒij.; Aquæ, ℥vj.; ft. sol. S. Take ℥ss. of each solution, effervescing every half hour.

The vomiting ceased. In the evening she had one scanty, dark, thin, evacuation. In the night of the 24th, she had fomentations. The general symptoms were so favourable, as not to require specific mention. On the 26th, suppuration was commencing in the wound, with some appearance of sloughing in the sac. On the 28th, the whole of the sac had sloughed away. The wound granulated, healed, and the patient was discharged cured on the 20th of September.

"This case," says Dr. Kirkbride, "is a very interesting one, from the unusual thickness of the sac, and from the sloughing of it, without any inflammation extending to the peritoneum generally, or even to the part contiguous to the sac, and from the *radical cure* effected by the loss of the sac, and by the firm adhesion established by the healing process. I visited the patient on the 27th of November, and found her enjoying good health; the parts are firm; she has never worn a truss, nor suffered any inconvenience in the parts."

### II. Tardy Union, and Absorption of the Callus of Fractures.

Case 3.—*Tardy Union—Friction of the Ends of Bone.*

"Dennis M'Fadden, æt. 30, labourer, of good constitution, but rather intemperate in his habits, was admitted on the 16th of May, 1834. While engaged, on the previous afternoon, at the Falls of the Schuylkill, assisting to raise heavy stones from a quarry, by means of a windlass and lever, by some means he lost his hold, so that the lever, in passing round, struck him violently upon the left arm, producing a fracture of the humerus, midway between the elbow and shoulder, throwing him a distance of ten feet, upon a pile of stones, and producing a fracture of the os femoris six inches from its inferior extremity. He has also received a severe contusion of the side, but no ribs were fractured. The arm was placed in pasteboard splints, and Desault's apparatus applied to the thigh. Ordered evaporating lotions; cups to the side."

Some blood appeared in the expectoration, and cupping was a second time resorted to. No further inflammatory symptoms followed; on the contrary, the patient grew pallid and lost flesh, and, by the 16th of August, no union of the fractured bones had taken place. On that day, friction of the ends of the humerus was made for the first time with considerable force, and continued for a few minutes without giving pain to the patient. This plan was persisted in daily for one week, when the parts began to be tender; during the next week it was done only every other day, and the pain of the operation increased on each repetition of it. The operation was continued till the 1st of September, after which it was not repeated. On that day, the femur was found to be uniting; on the 15th the patient was able to raise his leg without assistance, and the splints were in consequence removed. The humerus now appeared to be uniting, and, on the 30th, it was so firm as to bear the application of considerable force. The splints were retained upon the humerus until the 1st of November. On the 22d he was dismissed, having regained, in a great measure, the use of both the injured limbs.

Case 4.—*Absorption of the Callus.*

William Brum, æt. 21, carter, of good constitution, and enjoying good health, was admitted into the hospital on the 16th of April, 1834, with fracture of both bones of the leg, about five inches above the ankle, from the kick of a horse. There was no external wound, and a fragment of the tibia appeared detached. Suppuration took place, and a few small scales of bone passed through the opening. The separated portion of the bone united; suppuration ceased, and he was discharged on the 24th June. The ulcer had been well one week previous to his discharge, and during that time he had been walking about the grounds without the use of his crutches, his limb being perfectly firm. He again returned to the hospital on the 24th of July, with an ulcer over the seat of the fracture. He states, that after going out, he used his limb without inconvenience, but did not commence his usual occupation for a couple of weeks afterwards, and that after being employed for a few days, he found the limb more painful; he continued to use it, however, and to live freely. One week before his readmission, the ulcer broke out, but was of small size. On the 28th, a small spiculum was discharged, and the ulcer was reduced to a point. A probe introduced at this point, detected a portion of soft, bony matter. On the 15th of August, without any assignable cause, the ulcer commenced sloughing, and extended rapidly; his health began to suffer; he had chills, followed by fever; lost his appetite, and became rather prostrated. The sloughing was finally arrested by the application of caustic potash over the whole ulcerated surface; at this time it was observed that the callus uniting the fractured tibia was completely absorbed, and that the fragments were perfectly moveable, this separation of the bone appearing to occur simultaneously with the sloughing of the soft parts. The caustic was now applied to the exposed bone; considerable inflammation occurred a few days afterwards, but the parts soon took on a healthy action, and gradually improved, union taking place between the fragments of the tibia, so that by the 25th of October the ulcer had nearly healed, and the bone had become so firm that it was removed from the fracture-box.

Some ulceration over the anterior surface of the tibia existed up to the latter end of November, and small portions of bone were discharged from it. On the 1st of December, we find that no discharge had taken place for ten days previous to that time, and the patient was able to walk without crutches. The subsequent particulars are not stated.

Case 5.—*Absorption of the Callus.*

An omnibus driver, æt. 27, received a fracture of the left leg, about four inches below the knee, fracture of four or five of the ribs, and various severe contusions, in consequence of the wheel of the omnibus passing over him. Much swelling of the limb ensued, and fluctuation was distinctly felt in the groin. On the 30th of September, a collection of bloody serum just below the knee was opened; the swelling and fluctuation in the groin had disappeared, under the employment of a stimulating liniment.

By the 1st of October, the broken portions of bone had united; but considerable inflammation existed, from the knee to a short distance below the fracture. There was a discharge of serum, with oily globules floating in it, and some sloughs of the cellular membrane.

Oct. 12th. The swelling below the knee has gradually increased; it fluctuates slightly; a fluid may be pressed down from the knee, as far as the spot where the tumor appears to point, and which is opposite the seat of fracture. Upon opening the tumor, the only discharge was about a gill of dark, grumous fluid, with small portions of coagulated blood. For the last three days, a decided diminution of the firmness of the bone has been observed, and to-day the absorption of the callus appears complete, as the fragments are perfectly moveable. The limb has always been kept in the fracture-box.

Copious suppuration followed this opening, and was accompanied with some degree of fever. The suppuration, after a short time, diminished, and gradually firm union of the bone again took place. The report on the 1st December runs thus:—Bone continues firm—ulcer healing. No further record of the case is presented.

We would offer a few observations on these cases. The first differed from the other two in this respect, that, in it, union was long in occurring, while, in those, the union which *had* taken place was impaired or destroyed by the process of absorption. We have witnessed several instances of either sort. So far as we have seen, want of union, or a very tardy union, of fractured bone, has been, in all cases, the result of extreme constitutional debility. Of course, great disturbance of a broken bone is very likely to prevent its consolidation; but this is an extreme and an obvious case, and, in the greater number, this is not the cause of the deficiency of ossific matter. We will take the liberty of mentioning two cases, in illustration of what we have observed. When residing as house-surgeon in St. George's Hospital, we had under our care a groom, who had broken both bones of one leg. Little or no inflammation occurred, and all seemed doing well. The patient's health appeared to be good, and the limb was as quiet as could be wished. At the end of six weeks from the time when the limb was first put up in splints, we examined it, with the view of making the patient walk about on crutches. The union between the ends of the fractured bone was so soft as to permit the leg to be bent. We now made a very particular inquiry into the habits of the patient. We found he had been in the habit of drinking large quantities of porter. We ordered him a pint and a half of his favourite beverage daily. In the course of a fortnight, the limb was firmly united.

A house-surgeon received a middle-aged woman into St. George's Hospital, on account of a fracture of both bones of the leg, below its centre. She appeared to be healthy and tolerably strong, but she was corpulent, and of a very irritable habit. Some swelling and much spasm of the muscles of the limb ensued. The house-surgeon became alarmed, and freely bled the woman. She immediately felt exhausted, but the inflammatory and spasmodic symptoms subsided, and left the patient in a condition of some debility. Union never took place in the limb, and to this day she is unable to walk, even with the aid of an instrument, upon the limb.

Pregnancy notoriously prevents, in some instances, the union of a broken bone. The whole powers of the system are concentrated in the uterus. That this is the fact is evident, from the emaciation of the limbs in pregnant women, as well as by the cessation of the symptoms of phthisis, and other maladies, during the period of gestation.

Any thing which debilitates, any thing which powerfully withdraws the vital energies and actions to another part, and, finally, any thing which interferes with the process itself of union of the bone, may prevent that union from occurring. The same agencies are capable of doing away with the union when formed. But mere debility is less operative, in this instance, than in the simple prevention of the occurrence of consolidation. The most common cause of (to use an improper term) the dissolution of union which has occurred, is probably the supervention of disease in other parts of the body. We have seen several instances of this description. We recollect the case of a patient who had fractured the femur, near the trochanter. The fracture had apparently united, and the patient was going about on crutches. An abrasion on the affected limb gave rise to a severe attack of erysipelas, and the latter was succeeded by acute pericarditis. After death, the upper and lower portions of the femur were found to be perfectly moveable upon each other. The ossific matter must have been absorbed with extreme rapidity.

### III. Dislocation of the Os Humeri on the Dorsum of the Scapula.

*Case.* The patient, æt. 55, had been in the hospital, three months previously, for a dislocation of the os humeri into the axilla. On this occasion, the opposite os humeri was dislocated, so as to be placed on the dorsum of the scapula, immediately below its spine. We need not enumerate the characters of the displacement. The following modes of reduction were resorted to.

"The first attempt made at reduction was nearly that recommended by Sir Astley Cooper, and differing in no particular from that for a dislocation into the axilla. The patient being seated in a chair, the middle of a sheet was passed around the chest, and its ends fastened securely to a post opposite to the injured shoulder. The arm, just above the elbow, being covered with buckskin, another sheet was bound to it by means of a wet roller. The scapula was fixed by an assistant, making pressure against the acromion, the arm being nearly at right angles with the body, the pullies were attached to the last-mentioned band, and extension was made firmly and steadily for nearly half an hour, while at the same time, efforts were made to dislodge the head of the bone by rotation, but without success.

Mr. C. M. Coley of Bridgenorth, has recommended a different plan, which was also tried fully, without success, (*see Cooper on Dislocations, p.* 405). This consists in elevating the whole arm, and rotating it outward as much as possible, for the purpose of rolling the head of the humerus towards the axilla; when brought to resemble as nearly as possible a dislocation into the axilla, the head of the bone is to be retained in its situation, and the arm brought into a horizontal position, after which extension is to be applied as in ordinary cases.

Having failed in these efforts, while the patient was kept in the same position, an additional counter-extending band was fastened by its middle around that previously used, where it passed immediately behind the scapula; the other end of this second band was fastened to a firm point a few feet distant, so that when extension was again made, its direction, instead of being nearly horizontal and directly outwards, was considerably inclined inwards and forwards. The extending force was again applied, and in less than five minutes the displaced bone returned into the glenoid cavity, with a snap that was audible to the patient."

IV. INJURIES OF THE ABDOMEN.

*Case* 1. George Shivers, æt. 26, brickmaker, admitted August 26th, 1834. He is not very robust, but has generally enjoyed good health. About 4, p.m. of the day of his admission, he was injured by being accidentally thrown down, and receiving several kicks from a horse, under whose feet he had fallen. He was brought to the hospital one hour afterwards. The blows appear to have been received over the epigastrium and right hypochondriac region, where the skin is slightly abraded; he suffers extreme pain throughout the abdomen, and has also some pain in the head, on which are three or four trifling wounds. He had vomited before his admission, but no blood existed in the discharges; there has been no vomiting since he entered the hospital; the surface of the body generally is cool, that of the extremities strikingly so, and his pulse is very feeble and frequent. Stimulants and a grain of opium were given.

The patient slept little in the night, and next day there was intense pain in the lower part of the abdomen. There was tympanites, and some pyrexia. *Blister on the abdomen.* On the 29th the pain was less, the tympanites greater, the respirations were short, and deep inspiration impossible. In the evening, the skin and conjunctiva were become saffron-coloured, and the urine was similarly tinged. *Salines and opiates.* On the 31st, he had a loose discharge of blood from the bowels. On the 2d Sept. there was a similar discharge, and again another on the 6th. The yellowness of the skin diminished, as did the pain in the abdomen; the respiration was more free. The treatment consisted in mild diet, and oleaginous mixtures.

On the 9th, there was a return of pain, with vomiting, anxiety of countenance, and increased dyspnœa. These symptoms were relieved by effervescing draughts, with opium. On the 16th, a smooth tumor could be felt on the left side of the abdomen, two inches below the ribs. The pulse was feeble, and ranged from 100 to 130, the tongue was disposed to dryness, and was always red at the tip and edges. He was ordered calomel and opium. On the 26th, he had pain in his side, and inability to take a deep inspiration. A blister was applied with benefit. No great alteration in the symptoms occurred until the 22d of October. The gums had, in the interim, been slightly affected by the mercury. On the 22d, there was violent pain, succeeded by chilliness and fever. On the next day this was aggravated, with hurried respiration, and tenderness of the left side, near the edge of the ribs. Seventy leeches were applied to the side, with effect of producing a temporary mitigation of the pain. He became, however, delirious, pain became established in the right side, he gradually sank, and, on the 29th, he died.

*Dissection.* The peritoneal cavity contained about a quart of turbid, straw-coloured serum, with flakes of lymph floating in it. Pus was observed upon the membrane covering the intestines and upper surface of the liver; strong adhesions existed among the parts about the under surface of the liver and gall-bladder. The small intestines presented the Peyerian glands distinct, and slightly reticulated, but not ulcerated. Ulcerations were observed in almost every part of the large intestine, particularly in the lower two thirds. The ulcers were generally an eighth of an inch in diameter, with abrupt edges, and a cellular bottom. The liver was much enlarged and of a dull green colour throughout.

In the thorax, the right pleura contained about a pint of light straw coloured serum, with flakes of lymph floating in it. The left contained less than half a pint, with no lymph.

In this case it seems doubtful what amount or what exact description of injury was inflicted in the first instance. That no important viscus was materially affected would seem to be evident from the absence of proof of any such occurrence, on examination of the body after death. The patient died in consequence of chronic inflammation of the mucous membrane of the intestines and of the peritoneum. We cannot avoid entertaining the suspicion that more depletory measures at first would have been serviceable. We have seen many instances of abdominal injury, in which the symptoms were even more severe than in this, where vigorous, continued, and judicious depletion saved or seemed to save the patient's life. The prostration of strength, weak pulse, and cold extremities which frequently appear to forbid bleeding of every description, are commonly deceptive. We have seen the most extreme collapse where no important viscus was materially damaged. The surgeon ought always to act with caution, but still he ought to act.

*Case* 2. A labourer, æt. 43, admitted at 10 A. M. of the 25th October, 1834, the wheel of a cart having passed over the abdomen. When admitted, he complained of excruciating pain throughout the abdomen—the countenance was pallid and anxious—the skin cool and moist—the pulse very feeble and frequent—the respiration hurried. Stimulating applications were made to the extremities; a terebinthinate enema administered, and opium given pro re nata, to allay the excessive pain which he suffered. No re-action however was produced by the remedies; the abdomen soon became tympanitic; percussion was resonant in every part, and there was no distention of the bladder to be detected, although he had passed no urine since the accident. On the morning of the 26th, he discharged half a pint of liquid blood through the urethra, but no urine, and a catheter, which was afterwards introduced, brought away a small quantity of blood only. Death at 10 A. M.

*Dissection.* No appearances of any moment in the head or chest.

The abdomen was much distended, and contained a pint of liquid blood: while large effusions existed throughout the cellular substance on the posterior part of the abdomen, particularly in the region of the kidneys, and superior surface of the liver. This organ large, strongly adherent to the diaphragm; on its upper surface, under the peritoneal covering, exists an effusion of blood about three inches in diameter, immediately under which are found three fissures in the substance of the liver, with rough edges, each about one inch long, and one-fourth of an inch deep. In the substance of the liver, one-third of its thickness from the upper surface, is an isolated clot of blood of the size of a large almond. The right kidney was broken completely across with several fissures in its substance. The left kidney was also fissured. The bladder was contracted, and contained no urine.

### V. Sloughing about the Anus—Kreosote.

A labourer, æt. 42, admitted for dysentery, Aug. 16, 1834. The dysentery had existed for a week, and required little attention after the second day of his residence in the house. On the 18th, he experienced a sensation of weight and heat, and excessive tenderness on pressure in the parts around the anus. On the 20th, these parts were swollen and extremely painful. On examination, they were found red, and hard, and exceedingly tender when pressed—the pulse was frequent—the skin hot. Leeches, &c. were applied, but on the 22nd, a slough had formed on the left natis, and, an opening being made here, a large collection of offensive matter, with sloughs of cellular substance, was discharged. A probe could be introduced to the distance of five inches on either side of the rappé. After this a large slough separated on the left side, and subsequently other sloughs were detached, leaving an immense ulcerated surface extending from each natis four or five inches along the rectum, and as far as the scrotum on the perineum. Stimulants and astringents (for diarrhœa) were the means resorted to, but seems from the report that a solution of kreosote of the strength of 12 drops to a pint of water, was extremely instrumental in destroying the offensive odour of the discharges, and promoting a healthy appearance of the parts. On the 18th of October, the patient was discharged, the parts having perfectly healed.

It is probable that many other applications than the kreosote would have proved beneficial in the case before us. The compound tincture of benzoin is an admirable application to the sores left after extensive sloughing. The parts should be freely *washed* with this twice or oftener in the day, and subsequently lint dipped in the following ointment warm and melted, should be applied.

℞ Ung. elemi comp (Ph. L.).. ℥i.

Bals. copaib.............. ʒi.

Unguent. Sambuci....... ʒij.

M. ft. Unguentum.

This local management of sloughing sores we have found extremely beneficial. When the sore is granulating and disposed to heal, the solution of the nitrate of silver, or the red wash of Bates should be substituted for the compound tincture of benzoin as a wash, and the calamine cerate should be used as an ointment. General treatment applicable to the case is indispensable of course.

There is only one other case that need detain us. We give it, being short, unabridged.

VI. TEMPORARY LOSS OF VISION.

*Case.* "Ann Brady, æt. fourteen, was admitted on the 6th of March, 1834. She is a domestic in the family of a gentleman of this city, and states that when engaged in her duties a few hours before, she had been injured by a spark flying from the fire, and striking her left eye directly over the pupil; and that her vision immediately became indistinct. When admitted she complained of pain in the head; none whatever in her eye; she is unable to distinguish objects, but says there is the appearance of "a green cloud" constantly before her when her uninjured eye is closed. The appearance of the eye is perfectly natural; no change in the pupil, which contracts as usual on exposure to the light. Ordered cups to the head; mustard foot baths; cold applications to the eye; light excluded from it.

7th. To-day every thing before her is 'dark' when the uninjured eye is closed; she still has some pain in the head; none in the eye, the appearance of which continues natural. Apply thirty leeches to the temples; purge of calomel and rhubarb; cold applications and baths continued.

8th. Head-ache relieved yesterday by the leeches; has no power of vision; no change in the appearance of the eye; but now complains of deeply-seated pain in it. Ordered emplast. vesicat. 4 × 5, on back of neck.

9th. Blister drew well, This evening she is able to distinguish light from darkness; has no pain whatever; pupil natural; no appearance of inflammation. Purge with sulph. magnes.

11th. Since last report patient's vision has been gradually improving, so that now she is able to distinguish objects near her, or count one's fingers when held withia a few inches of the eye. Blister is kept open.

13th. Power of vision increasing; no pain; she is purged every other day.

17th. Patient is able to distinguish small objects across her room; but finds her sight bad in the evening; complains of pain in the head. Apply five cups to head.

19th. Vision nearly as good as that of the uninjured eye ; blister healing.

21st. Has recovered her sight perfectly.

26th. Discharged."

This concludes the Report before us. It contains, as we observed, some interesting facts, interesting to those who possess a genuine philosophical regard for their instructive study. One great aim which we have ever had before us has been to discourage theory and the love of theory, and to cherish that spirit of inductive reasoning which rests on observation, and finds in it wholesome and delightful mental nutriment. We are not without some hopes of having laboured not altogether idly or in vain.

---

### Sir Patrick Dun's Hospital.

### Recovery from a State of Fever almost desperate—Dr. Graves.

"I shall now speak of the case of Christopher Nolan, which I trust you have all watched with attention. When this man came into the hospital, his condition appeared to be completely desperate, he has, however, not only rallied, but is now convalescing rapidly. It is unnecessary for me to enter into a detail of his case, as I trust you have all observed it through its different stages; I shall only remark, that on his admission he was labouring under fever of the worst character, his body was covered with maculæ, he lay constantly on his back, and had low muttering delirium, was unable or unwilling to answer questions, his breathing was oppressed, his pulse rapid, small and failing, the powers of life awfully prostrated,—in fact, he was in a state of apparently threatening dissolution.

My first object was to rouse the sinking powers of the system, and with that view I adopted the following treatment. He was put into a comfortable bed, and heat was restored to the surface by diligently rubbing his trunk and limbs with warm flannel. I next ordered a succession of flying blisters to the neck, chest, and abdomen. I may observe here, that his chest was heaving, there was a general wheezing audible over the whole surface, and he had that peculiar livid expression of countenance and dusky hue of the skin, which indicate an imperfect aeration of the blood. With the view of stimulating the oppressed action of the respiratory nerves, I had two blisters applied, one on each side of the neck, above the clavicle ; after remaining on for two hours, these were removed, and two more applied over the supra-mammary region, then over the heart and right side of the chest, and lastly over the epigastrium. In addition to this he was ordered to have wine and chicken broth, and the following draught was prescribed, to be taken regularly every second hour until symptoms of re-action began to appear.

℞. Misturæ camphoræ, ℥j.,

Liquoris anodyni Hoffmani, ʒss.,

Spiritûs ammoniæ aromatici, ʒss.

Moschi grana octo.

In employing blisters in this case my object was to stimulate powerfully and in rapid succession the integuments of the neck, chest, and abdomen. This practice has in such cases been attended with very marked effects, and in ours proved extremely valuable. Its efficacy seems to depend, not on the discharge of serous fluid, or on any revulsive action of the blisters, but on the powerful stimulus applied to an extensive cutaneous surface. I may observe here, that, during the present epidemic, blisters have been one of our most efficient means of cure. In several bad cases I have blistered the nape of the neck, the chest, hypochondrium, and nearly the whole of the abdomen in succession, and often with remarkable benefit. In ordinary cases of fever, tenderness of the epigastrium, pain in the head, and derangement of the respiratory system, are best treated by the application of leeches, or even by the use of the lancet; but, in the present epidemic, I have observed that patients bear bleeding very badly, though practised at the commencement of the disease ; and the same rule applies, though with less force, to abstraction of blood by leeches or cupping-glasses. As far as my experience goes, local or general depletion should be resorted to with caution. I am not timid in the use of the lancet or leeches, but I have seen several cases of fever which terminated fatally, and these were chiefly cases where venesection had been performed at the commencement of the attack; and, with respect to leeching, I have found that those cases were very difficult of cure, in which, relying on my experience of former epidemics, I had leeched too freely for what I considered to be local congestion.

With respect to the general employment of blisters in fever, it is, I believe, a prevalent but erroneous practice to leave them on much longer than is necessary. I seldom let them remain on longer than four or five hours ; I speak here of flying blisters, which are applied successively to the nape of the neck, interscapular region, chest and abdomen. Sometimes two or three hours will be sufficient. It is true that a blister will seldom rise in this space of time; but, though you have no serous discharge, the moment the skin under the blister becomes red, every purpose is accomplished ; if the blister be removed, and the parts dressed with spermaceti ointment, it will rise in the course of a few hours. By acting in this way, two important advantages are obtained, you prevent the formation of bad sores, and obviate the risk of dysuria from the absorption of cantharides. It is in many cases extremely wrong to leave a blister on too long, and yet we see them frequently permitted to remain on for twelve, eighteen, and even twenty-four hours. If you excite the vessels of the skin by rubbing the part with a little proof spirit or oil of turpentine before the blister is applied, it will rise in three or four hours, if not it can be again applied. From a number of experiments made at the Meath Hospital, I have ascertained that the time necessary for producing the action of a blister is much shorter than is generally imagined. In some cases three hours, in some even two were sufficient, and in almost every instance the irritation of the cutaneous surface was effected in four hours.

I have stated that in this case we gave wine and chicken broth. With respect to the regulation of diet, I may observe that, in the present epidemic, a light nutritious diet may be prescribed at an early period of the disease. After the fourth or sixth day, we are in the habit of giving chicken broth, light beer, and even small quantities of wine. I do not speak here of chicken tea, as it is termed, but of broth of good strength and flavour. There is another point with respect to nutriment which I would beg leave to impress on your attention. When you give nutriment, be careful in observing the usual periods of meals. The space of time to which I limit the giving of chicken broth, jelly, arrow-root, and other mild articles of diet, is from eight o'clock in the morning to eight in the evening. Always make it a rule that your patient shall take nutriment within the space of those twelve hours during which he is accustomed to take his meals when in health, and allow him nothing but mild diluent fluids during the night. I am persuaded that I have seen much benefit derived from following this simple plan.

A few words in conclusion with respect to the stimulating draughts which we employed in this case with such remarkable effects. During the prevalence of those doctrines which attributed all fever to local inflammation, diffusible stimulants fell into neglect and disuse; but, since our knowlege has been rendered more certain and fixed by the results of a truer pathology, we have learned to estimate their real value. I do not by any means exaggerate when I say that I have seen many lives saved by a combination similar to that employed in the present case. Where there is great prostration of the powers of life, oppression of the nervous functions, and low, muttering delirium, I do not know of any remedy which can be prescribed with more advantage. You will, of course, while employing medicines of this description, attend to the state of the bowels.

In the mixture we have ordered, you will perceive that the principal ingredient is musk. Musk exercises a stimulant effect on the nervous system, without having any tendency to produce cerebral congestion or coma. Unlike those remedies which powerfully affect the nervous system, it does not produce intoxication or narcotism.—Hence it is that musk proves such a valuable stimulant in cases such as I have described, where there is reason to apprehend congestion of the brain. At the same time that you prescribe musk, you should assist its action by blisters judiciously applied. They may be applied along the sides of the neck, or over the chest, to excite the nerves of the heart, lungs, and diaphragm; or you may apply them between the shoulders and to the back of the neck, where your object is to act on the brain and spinal marrow. In cases like this, the best places for applying blisters are the neck, region of the heart, epigastrium, and spine. The blister should be small, and you should make up for their want of size by successive applications.

I have only to add that the treatment adopted in this case succeeded in again rousing the almost suspended powers of the system, the patient rallied, and his fever assumed a much more manageable aspect. I was obliged, however, to have recourse to the tartar emetic mixture with opium, in order to produce sleep. This completed his cure; from the time of its exhibition every thing went on well."—*London Med. and Surg. Journal.*

---

BRISTOL INFIRMARY.

OSTEO-SARCOMA OF BOTH JAWS, FOR WHICH AMPUTATION AT THE JOINT WAS PERFORMED. By W. HETLING, Esq. Lecturer on Surgery, and Surgeon to the Infirmary, Bristol.*

This case forms the subject of a pamphlet of 63 pages. It appears in every way creditable to the daring surgeon who was the operator, and is the narrator of the particulars. We will give those particulars, before we advert to any of the observations of Mr. Hetling.

*Case.* Margaret Hunt, æt. 23, of a scrofulous habit, sallow complexion, and with a very feeble and limited intellect, was admitted into the Bristol Infirmary, 27th January, 1831, with a large and prominent tumour of the left side of the face, extending over both jaws, and appearing to fill up the cavity of the mouth; elevating and pushing before it the integuments of the cheek. The resistance which the cheek offered, repressed and flattened the surface of the tumour, and in consequence of this resistance, a portion of its lobulated structure was forced towards, and partly protruded at, the angle of the mouth between the lips. The integuments, also, both of the nose and eye, were disfigured from the same cause; forming, together, a very striking and fearful deformity of the face. The tumour extended from the upper to the lower jaw, to the latter of which it adhered so firmly, as to render it completely immoveable, so that the patient could not masticate, and could scarcely articulate, being only enabled to answer questions put to her, by indistinctly mumbling yes or no. In this state she was compelled to live upon fluids, and even these were with difficulty swallowed, deglutition being much impeded by the pressure of the tumour upon the internal part of the mouth.

Upon accurately examining the internal part of the mouth with the finger, it was ascertained that the tumour originated in the superior maxillary bone; and that in its progress it had gradually extended backward into the palatine portion of the mouth, and been pressed forwards and downwards upon the lower jaw by the action of the cheek; where, by its long continued growth and increasing pressure, it had produced extensive absorption of that bone. The alveolar border and processes of both jaws, were nearly all removed on that side of the face; in the upper jaw not a tooth was left; in the lower only a few remained, loose and buried in the

* We have been accidentally prevented from noticing this interesting case at an earlier opportunity.—*Eds.*

medullary matter of the tumour. One rather unusual, and, with a view to an operation, favourable circumstance was, that the tumour prevented the closing of the mouth in consequence of its insinuating itself between, and adhering to both jaws, so as to render them fixed, to a certain degree, asunder; this circumstance afforded the opportunity of exploring, by the finger, the inside of the mouth, by which means it was ascertained that no adhesive, ulcerative, or fungating process had taken place between the surface of the tumour and the cheek, although they were in close contact, and, as may be supposed, firmly pressed together. The fact of finding the cheek free, healthy, and unadhering, so as to allow the finger to pass between it and the tumour, and over its surface, quite up to the articulation, was an unexpected feature amidst such a mass of disease, and contributed to encourage the prospect of the projected operation. The elastic properties of the cheek had caused it gradually to yield, and accommodate itself to the growth of the tumour, in a manner similar to that which the parietes of the abdomen so remarkably exhibit in cases of dropsy. The glands of the neck were found free from disease.

Externally, the tumour gave the sensation of firmness, from the tension of the cheek over it; but internally it felt soft and yielding, about the consistence of brain. The shape of the tumour may be conceived from the above description; with respect to its size, it was about equal to a large orange.

The friends of the patient stated that the swelling had commenced after exposure to cold, when the girl was between eleven and twelve years of age. Several of the teeth on the affected side had dropped out. Latterly there had been frequent and copious bleedings from the mouth.

On consideration of the circumstances of the case, Mr. Hetling very judiciously determined to operate, and this view was supported by his colleagues in consultation. The mode of operating must be stated in the operator's words. We should state that a few days prior to the operation, he again accurately examined the parts, and removed the few remaining teeth out of his way.

"Feb. 16.—The patient was laid horizontally on the table, on the right side, the head moderately elevated on a firm pillow, so as to place the cheek in the most favourable position for *the operation.* Two fingers were introduced at the angle of the mouth, between the tumour and the cheek, in the direction of the articulation; the integuments were then freely slit up between them to the lobe of the ear; another incision was then carried from the infra-orbitary ridge across the centre of the first incision, down to the angle of the lower jaw. It was here necessary to tie some branches of the facial artery, which bled rather freely. The flaps of the crucial incision were then reflected, which fully exposed the external and irregular lobulated surface of the tumour, and afforded, also, the opportunity of tracing its base and attachments. The base of the tumour was found to occupy the palatine and maxillary portion of the upper jaw, and in its extensive growth its head had been forced down and attached to the ridge of the lower jaw, nearly as far as the symphysis, extending along the whole of the alveolar border nearly in a horizontal line from the mental foramen to the condyloid process, the whole of which portion was discovered to be either absorbed or in a state of caries from the long-continued pressure of the tumour. In fact, the tumour had so worked its way across the lower jaw, both inwards and outwards, that it was found buried in its substance, and, consequently, absorption of its body had been going on for some time, on both sides of the bone. The substance of the tumour was next separated by the knife and fingers, from its base and adhesions; this occupied a considerable time, in consequence of its various sinuations, as it had penetrated in every direction where it could find access. When this was effected, an extensive irregular surface of bone was found in a state of caries, extending, in the upper jaw, from the pharynx across the palate to the malar bone. Not the least vestige of the thin walls of the antrum remained. Fortunately the floor of the orbit was left uninjured. With the assistance of Liston's bone cutter, small saws, &c. every portion of diseased bone was taken away that could be safely removed, and the general surface scraped, as carefully as possible, with the knife, it being intended, finally, to apply the actual cautery over the whole plane of the diseased bone. Having accomplished this tedious and difficult part of the operation, ample room was found for amputating the lower jaw at the articulation; caries having extended, as before stated, from near the symphysis along the whole of the upper margin to the joint. This extensive line of bone was then sawed off, except the condyloid process, which was afterwards easily disarticulated, and removed with Liston's bone cutter, having first divided the fore part of its capsule, and also the temporal muscle from the coronoid process. This important and generally intricate part of the operation, was facilitated by the previous removal of all the teeth and alveolar processes.

During the operation, no alarming hæmorrhage occurred, and after the branches of the facial artery had been secured, but little interruption was experienced from loss of blood."

Mr. Hetling was induced by the gentlemen around him not to use the actual cautery, an omission which he afterwards regretted. The flaps of the cheek were brought into apposition by three sutures. On the fourth day the external wound had united. In a fortnight she walked about the ward convalescent—in a month, she masticated almost as well as usual with the other side of the mouth—and in three weeks after this (April 5th) she was dismissed quite restored to a healthy appearance. She was directed to return to the Infirmary, if any appearance of the disease should occur.

On examination of the tumor, its lobulated shape had been mostly destroyed, in consequence of considerable portions having been necessarily torn apart by the finger during the operation. Its substance throughout possessed the common homogeneous structure of medullary matter, being composed of irregular masses inclosed and separated from each other, more or less completely, by thin membranous septa, bearing, in colour and consistence, a near resemblance to brain. On cutting it into slices, a few blood-vessels were divided, from the orifices of which oozed small portions of blood. In fact, it possessed all those anatomical appearances usually characterising medullary sarcoma, with which every practical surgeon is familiar.

Mr. Hetling offers many interesting observations on the case before us, and on the great confusion that prevails respecting the nomenclature and the classification of morbid growths from bone. He expresses his opinion, as many other surgeons have done, that the term osteo-sarcoma is a bad one, and agrees with Mr. Lawrence, and indeed with most scientific surgeons, in the idea, that tumors of bone should be named from their specific characters, and not in this vague and indefinite fashion. But passing over this portion of Mr. Hetling's paper, which, though good, presents no feature of novelty, we may notice his description of the disease as it affects the jaw.

" The term osteo-sarcoma of the jaw," he observes, " is employed by most authors to designate a morbid product, the distinctive character of which consists, more or less, in its resemblance to brain, fat, &c. It is true that the species of tumour which passes under this designation is sometimes found to consist of other materials either more or less hard, pulpy, or soft, such as fibro-cellular, fibro-cartilaginous matter, coagulated blood, serum, &c.; but these varied substances are generally to be found rather as intermixed with the original medullary structure of the tumour, than essentially belonging to it; and may be more properly considered as superadded, or converted materials, arising from occasional inflammation excited in the parts, after the tumour has been formed, than as composing its original and primary formation. I therefore conceive that under the term osteo-sarcoma, according to the present nosology of all English practical surgeons who have recorded cases of this nature, are included all those depositions which pass under the common epithets of osteo-sarcoma, cancer of the alveolar membrane, soft cancer, medullary exostosis, fungus medullaris, medullary sarcoma, medullary cancer, encephaloid, cerebriform, fibro-cellular, fibro-cartilaginous formations, &c. &c. These malignant productions admit of almost endless modifications."

"From this representation, and under our present state of knowledge, osteo-sarcoma may perhaps be advantageously separated into two great divisions; the first to be considered as accidental from a local external cause; the other as arising independently of the direct influence of any local cause, but owing to some great change in the system, which influences the important functions of secretion and deposition, as constituting what is termed an idiopathic disease. These again may be subdivided into the hard, or fibrous, fibro-cartilaginous, fibro-cellular; and into the softer, or medullary, pulpy, and fungus medullaris, &c. &c. Nothing is more difficult than to trace the origin and cause, or to estimate the kind or the extent of the changes which take place in most of the varieties of tumours; changes which are often scarcely perceived, while they are making considerable progress."

We think there can be no doubt that great differences exist in the physical characters and the quantum of malignancy of tumors arising from bone, and we think also that as little doubt can be experienced respecting the difficulty that attends the diagnosis of these varieties of morbid growth, and the power they possess of mutual convertibility. We have seen a fibro-cellular morbid growth arising from the lower extremity of the femur, succeeded by genuine medullary tubercles in the lungs. Some of the growths from the superior or inferior maxillary bone approach very closely to, and others recede almost as widely from the characters of medullary sarcoma. In proportion as they do approach that formidable type of malignant growths, the chances of success from their removal diminish, and the surgeon is less justified in recommending his patient to submit to a cruel, because useless operation. An accurate study of the anatomy of these morbid growths from bone, and a careful investigation of their respective symptoms are therefore indispensable on the part of the surgeon.

The following is the general description presented by Mr. Hetling.

"In general, the origin of osteo-sarcoma of the jaw, may be traced to unsubdued or mismanaged local inflammation, supervening upon a scrofulous disposition, from whatever cause produced, whether cold, a blow, or mechanical injury to the jaw or teeth, stumps, &c. or other sources of excitement, which occasion the inflammation to extend to the medullary membrane, periosteum, or the internal and cellular structure of the bones of the face. It may attack any of the tissues of the body, but the vascular periosteum that lines the thin walls of the antrum maxillare and the alveolar depressions of both jaws, appears to have the greatest disposition to generate this disease, as it is generally found invested throughout with that fine membrane. The palate bones which form part of the roof of the mouth, the floor and side of the nostrils, the floor of the orbit, are also liable to this disease; but, in most instances, within my observation, these parts become involved in this disease, owing to its spreading by consent of parts, and its contaminating presence in the alveolar membrane, or the antrum. Its growth may be either slow or rapid; it attacks all ages, but chiefly occurs from infancy to forty years of age. Both sexes are liable to it. Its growth is not generally attended with severe pain, unless where it happens to press upon a nerve, or is closely confined within the structure of bone, exciting inflammation and ulceration of that tissue, and thus producing caries. The pain is therefore uncertain and inconstant; sometimes severe, at other times scarcely perceptible, but never of that incessant lancinating, stinging, burning, darting kind which is common to cancer. The neighbouring glands are generally unaffected. Excavation of the bone takes place in proportion as the internal formation of the tumour increases, and excites absorption, first of its cancellated, and ultimately of its more solid osseous structure. Every thing seems to give way to this growth; and the destruction it occasions is immediately supplied by its own development. At its commencement, this malignant growth is bound by the parietes of the bone, which at length yield to absorption from unnatural pressure, and then the tumour, becoming freed from its confinement, spreads in the least resisting direction: this is exemplified in the above case, by the lobulated protrusion at the mouth. The anatomical character and composition of the tumour is of various degrees of consistence and structure. But the principal pathognomonic appearance which this morbid product presents, is, in general, its resemblance to brain, both anatomically and chemically. This appearance is, however, modified, as in all other tumours, according to the locality of its seat or tissue, and the latent predisposition of the patient. This may be said to be its generic character; but graduated deviations occur both in structure, consistence and colour. In some instances, the consistence of the mass is fatty, soft and brainy, or fibro-cellular. In its more advanced stage, the lobes of the tumour sometimes burst, and send out an excrescence or fungus that sprouts more or less exuberantly; the teeth loosen, and fall out occasionally; a fetid, bloody discharge flows from the mouth, sometimes attended with frequent hemorrhage. Hence in this stage it has sometimes been denominated, and confounded with, fungus hæmatodes; but this bleeding, in general, is only occasioned by ulceration and rupture of some of the vessels that nourish and perambulate its substance. I do not deny that fungus hæmatodes may supervene upon this as well as in any other class of tumours; but I wish to guard against the conclusion, that every tumour that bleeds is to be considered a case of fungus hæmatodes, which is a disease of peculiar organization of vessels.

This is the single or simple description of osteo-sarcoma, but, like every other morbid growth, it may occasionally exhibit, as supervening diseases, the production of carcinoma, melanosis, and fungus hæmatodes, in the same organ or tissue, and in the same diseased mass."

Mr. Hetling's remarks on the diagnosis of osteo-sarcomatous growths may, we think, be omitted. He takes true scirrhus, and points out the general and familiar differences between it and osteo-sarcoma. But he need not have raised this man of straw. No one confounds scirrhus and osteo-sarcoma —the real difficulty lies in separating the latter from medullary sarcoma.— We fear that too often there is no separation—that frequently they are one and the same disease. There are indeed cases in which the general characters of the tumor, its very slow progress, and the condition of the patient, proclaims its fibro-cellular nature. But these are rather the exceptions than the rule—and even in these cases, flattering as they appear, the characters of the most inveterate malignancy are not unfrequently developed, and operations are followed by the appearance of unequivocal malignant growths in other portions of the body. The surgeon in his diagnosis must be guided by the circumstances of the case before him, and no general rule can compensate for the want of experience and of judgment on his part.

Mr. Hetling occupies himself with the consideration of the propriety, or otherwise, of securing the common carotid artery previously to removing the morbid growth. The influence of facts, and the force of reasoning, seem equally to convince him that the application of a ligature on the artery in question is in general unnecessary and injurious. We perfectly agree with Mr. H. The expedient is a clumsy, inefficient, and dangerous one, and few cases can occur, in which a bold and judicious surgeon would be temped to resort to it. The best plan is to secure the vessels that are wounded, and only to tie the common or external carotid in case of imperious necessity. Mr. Hetling cites a number of cases, and enters on rather an elaborate investigation of the question. We shall merely introduce his conclusions.

"This comprehensive anatomical and surgical survey of cases and facts, leads, it is presumed, to the important conclusion, that tying the primary carotid artery is to be considered as unadvisable, as a preliminary step, in the removal of tumours about the head, neck, and face; and that such a proceeding is only to be recommended as positively necessary in attempting the cure of aneurism of either of its trunks.

This deduction is evidently at variance with the prevailing practice amongst several of the first surgeons of the present day; and, in consequence of the influence of their opinions and example, it has become nearly a common rule, whenever a tumour about the neck or face, of any magnitude, is to be extirpated, to proceed, unhesitatingly, in the first instance, to tie the common carotid artery. It is to guard against the prevalence of this, in some instances, inconsiderate, and too general practice of causing a patient to undergo two severe and dangerous operations, that so extensive an analysis of facts and surgical operations has been entered into; the real and sole design of which is to bring before the profession this, as it may be termed, double operation, on an extensive scale, and in all its bearings, and with the hope of obtaining and establishing, from a fuller and larger collection of facts and opinions, the most accurate principle of practice, with respect to the single or double operation."

The last point that engages the attention of our author is the plan of tying the principal artery going to supply a tumor, in order to procure the wasting of the latter. Mr. Hetling seems unsettled in his notions of the value of this method of proceeding. For our own parts, we doubt if much will be obtained from it. The cases in which we may fairly expect most are those of affections of the blood-vessels themselves. Yet the operation is of doubtful, very doubtful utility, even in aneurism by anastomosis.

With the following novissima verba of our author, we quit for the present this interesting surgical subject. He thinks, from various facts and considerations, that he may safely be permitted to conclude:—

"1. That the preparatory operation of tying the primary carotid artery, is not only, as a general principle, unnecessary, but should be discountenanced as a proceeding of great, and, indeed, of far greater danger than the second operation; namely, that of removing the tumour, with the necessary portions of diseased bone, in cases of osteo-sarcoma of the jaw.

2. That there is every pathological reason for concluding that, in the great majority of cases that have been referred to, where the double operation has been performed, the result has proved fatally detrimental to the patient; and that this is solely to be attributed to the superadded operation of tying the common trunk.

3. That all the most extensive and extreme cases that have been *singly* operated upon, that is, without tying the carotid artery, have escaped a fatal hæmorrhage, and have, at least, recovered from the effects of the operation, even where the final event has been fatal.

4. That where the double operation has been performed, it has, in some instances, been followed by consecutive hæmorrhage, of which the patient has died, thereby causing the fatal event which it was designed to avert.

Lastly, not only is it proved that the removal of the tumour, with the diseased portions of bone, is an inferior and less dangerous operation than tying the carotid artery, but that it is an operation that may be extended to both jaws and bones of the face with less constitutional disturbance than has been apprehended; in fact, that almost the entire of one side of the bones of the face may be safely amputated, and the patient still be able to perform the functions of mastication and articulation, accompanied with but slight deformity."

We think that Mr. Hetling would do well to acquaint the profession with the termination of the case upon which his instructive pamphlet is erected.

---

### Guy's Hospital.

### Renal Dropsy.

Mr. Anderson, lately clinical clerk at Guy's Hospital, read a paper before the Senior Physical Society of the same hospital on renal dropsy, which was afterwards inserted in our weekly cotemporary, the Medical Gazette. It is now published in a separate form, and we purpose taking a short notice of it here. The author observes that dropsical effusions, connected with albuminous urine, are induced by several causes—by scarlatina—by abuse of mercury—gout—abuse of tonics—disorders of the digestive organs—but especially by exposure to cold and wet—and by inordinate ingurgitation of spirituous liquors. Most of the renal cases that came under his observation in the hospital proceeded from these latter causes.

"The sedative effects of cold and consequent deranged function of the skin, and the debility of constitution and irritation, induced by spirit-drinking, through the medium of the stomach, are well known. These causes predispose the kidney to suffer: this irritable organ sympathizing with the skin, a change in the renal secretion takes place, and, owing to checked perspiration, a vicarious discharge in the kidneys is produced; they become functionally deranged, and albumen appears in the urine; deranged function leads to disorganization; serum is effused first in the cellular membrane of the face, feet, and ankles; then gradually extending upwards to the thighs and abdomen; and the aching pain in the loins, the severe throbbing and lancinating pain in the head, and apoplectic tendency; the hard pulse, dry unperspirable skin, and albuminous, dingy, and sometimes bloody urine, fully characterize and establish a true and exquisite case of renal anasarca."

Mr. A. then proceeds to detail the case which forms the basis of his paper. Fred. Crown, aged 47, a stout and well-formed man, was admitted, Dec. 3d, 1834, under the care of Dr. Bright. He stated that he had been ill for six months—and had been subject to rheumatism for some years. He attributed his present illness to an attack of rheumatism. For four months he had laboured under anasarca, appearing first in the face and ankles, and afterwards extending to various parts of the body. He was an intemperate man, and much exposed to atmospheric vicissitudes. The most prominent symptoms now were—pallid and sallow countenance—dull pearly eyes—eyelids œdematous—lower extremities anasarcous, as also the scrotum—no ascites—lancinating pains in the head—oppression at the epigastrium—pain in the region of the kidney—palpitation and dyspnœa on using the least exertion—action of the heart laboured, and somewhat indistinct—pulse 104—tongue dry—skin unperspirable—urine of a pale straw colour, coagulable by nitric acid and heat—specific gravity 1·015—gouty and rheumatic pains in the hands.

### Diary.

"℞. Vin. Ant. ♏xx. ex Jul. Ammoniæ Acetatis, ℥j. sextâ quâque horâ.

℞. Pil. scillæ c. Hyd. gr. x. omni nocte.

December 4th.—Pains very much increased; pulse 100, with some jerk.

Mittatur sanguis e. brachio ad ℥xij. Applicetur Cataplasma lini lumbis bis in die. Omittantur Pilulæ. Pergat Mistura.

5th. Blood drawn slightly buffed; pain in the loins relieved; that in the head increased; bowels rather confined.

C. C. nuchæ, ad ℥x. ℞. Ol. ricini, ℥vj. statim.

6th. Going on more favourably; symptomatic pains better; gout and rheumatism troublesome; urine rendered only opalescent by heat; very coagulable, however, by nitric acid.

Pergat.

11th. Pains in head and loins much increased; dyspnœa very severe; skin dry; pulse quick and hard, with some jerk; urine about natural in quantity, acid, and very coagulable before boiling. Mr. Stocker saw him, and ordered the following:—

℞. Pil. Camb. comp. c̄ Cal. āā gr. iij. statim. C. C. lateri dextro, ad ℥xij.

12th. Pains rather relieved; skin dry; pulse incompressible; rheumatic pains more severe. Dr. Bright ordered the following:—

℞. Tr. Camp. comp. ʒss. c̄ Vin. colchici, ♏xx. et Liq. ammon. acet. ℥ss. ex Mist. Camph. ℥j. ter die.

℞. Hyd. submur. gr. j. sextâ quâque horâ. Admov. Emp. cantharidis sterno.

15th. Pain in loins increased; dyspnœa great; tongue dry; skin hot; pulse strong; urine very coagulable, but rather diminished in quantity; bowels rather confined; feels nauseated from the medicine.

℞. Mist. Magn. c̄ Mag. sulph. ℥jss. pro re nata sum.

℞. Sp. æth. nit. ʒss. c̄ Liq. ammon. acet. ℥ss. ex Mist. camph. ℥j. ter die.

To this latter, Mr. Stocker added on the following day—

Acet. scillæ et Tr. Hyoscyami, āā ʒss.

22d. Has been a little improving up to the present time; but to-day the symptoms have recurred with their usual violence; great soreness of the loins complained of, and pain extending down the groin to the scrotum, with slight retraction of the testicle; also, a soreness and dryness in the throat, affecting deglutition, and causing a cough: these feelings probably owing to œdema of the glottis. Right hand puffy and œdematous; great thirst and heat of skin; tongue dry; pulse quick and incompressible; bowels regular; urine of the same appearance, acid and coagulable.—Pergat.

27th. Œdema of upper extremities, and particularly of the right hand, very much increased; pain in the throat, and difficulty of deglutition,. very severe.

Admov. Emp. cantharidis gutturi.

℞. Pil. Scillæ comp. gr. v. ter die.

℞. Liq. ammon. acet. ℥ss. ex Mist. camph. ℥j. ter die.

30th. Cough very troublesome; expectorates some viscid mucus, stained with blood; throat very sore; œdema of right hand increased; urine in good quantity, and very coagulable.

Pergat.

Jan. 5th, 1835. Pain in the head has continued very severe; breathing rather easier; skin dry; tongue coated; pulse quick; slight difficulty in passing his urine, which is of the same character and appearance.

℞. Mag. sulph. ʒss. c̄ Vin. ipecac. ♏v. ex Inf. gent. co. et Inf. rosæ co. āā ʒvj. sextâ quâque horâ. Omittantur alia medicamenta.

He continued during the next week rather to improve, though the throat was very sore, and the urine very coagulable. A few days afterwards (the day preceding his death), he was observed by Dr. Bright to be better in his general health; but, towards evening his manner of speaking seemed rather singular and unusual; his breathing became more oppressed during the night, and he would not answer when spoken to. These symptoms and appearances continued to increase during the next morning, when, about half-past 11, a.m. he suddenly went off into a kind of apoplectic fit; his breathing became stertorous, eyes protruded, and pupils contracted; he foamed at the mouth, had convulsive startings, and the face was of a yellowish tinge.

He was ordered to be cupped behind the ears, a blister to be applied to the shaved scalp, and one grain of calomel to be taken every four hours.

He rallied, but had four more attacks of the same character, and a last one at 3 o'clock, when I was with him. The pupils were then dilated; the breathing was stertorous, and at long intervals; the face and hands were bedewed with a cold, clammy perspiration; and in a quarter of an hour he died.

An inspection took place twenty-two hours after death. For the following minute and interesting account of the post-mortem appearances I am indebted to Mr. Sibson:—

There was a general state of anasarca present all over the body, penis, scrotum, upper and lower extremities. On cutting into the cavity of the abdomen, which was exceedingly tumid and tense, upwards of a gallon of clear, slightly yellow fluid escaped. Liver appeared to be remarkably healthy; gall-bladder nearly filled with dark greenish bile. Spleen small; numerous white opaque spots on its surface; substance healthy; Pancreas rather small; healthy. The small intestines were rather blanched on their surface; their appearance gave rise to the idea of their being invested with a false membrane, from which, however, they were quite free. The stomach: towards the cardiac extremity, and along the greater and lesser curvature, the mucous membrane was soft, and easily separated from the middle coat. The mucous membrane of the pyloric extremity, for two or three inches from the orifice, was hard and granular; the pylorus itself was nearly half an inch in thickness, dense and white in its structure. The duodenum, for two or three inches at its commencement, had its mucous membrane hard and minutely granular. The rest of the small and the large intestines appeared perfectly natural. The left kidney was of little more than half the usual size, slightly lobulated on the surface, and of a much firmer texture than natural. The investing cellular membrane adhered pretty firmly to the proper tunic, below which were seen eight or nine cysts about the size of a pea, but varying a little in magnitude, and filled with clear fluid. One cyst of the same size was opaque. The proper tunic was readily separated from the surface of the kidney, which was covered with minute white granulations, of the size of small pins' heads; they were clearly contrasted with the light brown colour of the cortical texture. There were one or two white spots, about half the size of a silver penny, near the entrance of the vessels. On cutting into the kidney, its substance was found to be hard, and similarly granulated to the surface in its cortical part. One or two white spots were seen on the tubular substance; and the cortical part was exceedingly narrow, the tubular structure appearing to be of rather more than its natural dimension. The infundibula and pelvis presented nothing remarkable. The vessels of the right kidney were injected; the veins with blue, the arteries with red size. The veins presented numerous stellæ on the surface. The arterial injection shewed itself in numerous small packets in the cortical substance; but the whole of the tubular, and greater portion of the cortical, part remained uninjected. The right kidney was similar in every respect to the left. The bladder was filled with urine.—Thorax: the cavity of the pericardium contained about half a pint of pretty clear, straw-coloured serum. The pericardium itself presented nothing remarkable. Heart: the cavity of the left ventricle was about four times its usual size; it was firmly contracted, and presented very great hypertrophy, the walls being about three-fourths of an inch in thickness. The mitral valve and semilunar valves of the aorta were quite healthy. The calibre of the aorta was nearly double its usual size. The right ventricle was considerably dilated, and its walls were thinner than natural. The right lung was free of pleuritic adhesions; it was crepitant throughout, and natural. The left lung was considerably congested and œdematous, especially at the posterior part; it was crepitant throughout. The pleura pulmonalis, on the left side, was universally adherent to the pleura costalis. The cavity of the pleuræ contained about a pint of straw-coloured fluid.—Head: there was very considerable sub-arachnoid effusion on the surface of both hemispheres and at the base of the brain. The arachnoid presented small and slight opacities in numerous points. The pia mater was readily separated from the convolutions, and from their surface a thin pellicle of cineritious matter could be easily peeled off. The lateral ventricles were distended with about two ounces and a half of clear fluid. The carotids were at points opaque, and semi-cartilaginous.

On cutting into the knee-joints, they were each found to contain about an ounce and a half of semi-opaque synovia, mixed with loose flakes of a white matter, having a gritty feel. The articular surfaces of the cartilages of the femur, tibia, and patella in both joints, were covered with a white gritty substance, and the synovial membrane had imbedded in it, at various points, masses of a calcareous concretion, the largest of which was about an inch in length, and half an inch in thickness. The cartilages were of nearly double their usual thickness, much less firm in their texture than natural, and of a brownish colour. The elbow-joints contained a similar secretion; but the surface of the shoulder-joints was quite smooth, as also the phalangeal articulation of the right great toe.*

A portion of the effused fluid from the different cavities was analysed by Dr. Barlow, who has been kind enough to favour me with an account of the process:—

'An aqueous extract was obtained from each of the fluids, and from this extract an alcoholic one was procured, absolute alcohol being used for the latter purpose: all the evaporations were conducted at a temperature not exceeding 200° F. Of each extract a syrup was made, by the addition of a few drops of distilled water; and to each of these syrups were added a few drops of strong nitric acid. The syrup from the effused fluid of the brain, as also that of the abdomen, yielded within two hours a considerable crop of foliaceous pearly crystals, of an arborescent form. That from the pericardium, as also that from the pleuræ, yielded a very small crop of crystals, after a much longer time.'

* "Preparations of the injected kidney and diseased joints are carefully put up, and preserved in the museum of the hospital."

Dr. Barlow has strong reasons for believing that these crystals were nitrate of urea."

Mr. Anderson selects a good resumé of the opinions of modern medical men respecting the pathology of the disease. The testimony of Dr. Bright is, perhaps, the best. He observes, that he has never yet examined the body of a patient dying of dropsy, attended with coagulable urine, in whom obvious derangement of structure was not detected in the kidney. The testimony of Drs. Blackall, Tweedie, Darwall, and others, is not very dissimilar. The heart is very frequently found to be affected organically. The case which we published last quarter of Mr. Ward, in the City, is a striking illustration. This connexion throws light on the apoplectic tendency of cardiac and renal diseases.

"With regard to the treatment,—this must be directed with a view to restore the healthy function of the kidney, and to guard against any inflammatory affection, or apoplectic seizure, that may and does arise. That it is often unsuccessful, is but too true. We must, however, remember that the coagulable state of the urine may exist long before the anasarca shews itself: the patient's attention is not arrested until this latter affection occurs; and when at length our remedies are administered, the kidney is far advanced in disease. Does it not, therefore, behove every practitioner to examine carefully, in every suspicious case, the state of the urinary secretion? General and local depletion, and a strict antiphlogistic regimen, are the principal indications of cure; for (as Dr. Bright observes) there is reason to believe that a state of great congestion, perhaps an actual process of slow inflammation, exists in various internal organs, and particularly in the kidneys, where it probably lays the foundation for their future disorganization. Hydragogue cathartics act well—as jalap, elaterium, &c.; but we must be cautious not to purge too much, for there is a fear of abrasion of the mucous membrane of the intestines; perhaps also a danger of exciting or increasing inflammatory action in the kidney. Digitalis may be employed, with caution. Supertartrate of potash, from its gently purgative and diuretic property, is of great service; but all stimulating irritating diuretics are to be avoided, and we must act gently on the skin by saline diaphoretics. The loins should be surrounded with a large linseed poultice, which acts as a fomentation to the part, and will be found a very soothing application. Milk diet is the best. Tonics are indicated by the debility occasionally present; and, in the more chronic forms, Dr. Bright is inclined to think they may be of benefit. Dr. Blackall also speaks of the use of tonic remedies. The occasional alkaline property of the urine in confirmed renal disease, in connexion with the *absence* of albumen, as before alluded to, would seem to indicate the employment of an alkaline remedy. Dr. Bright, with this view, tried the liq. potassæ in one case, but not with much apparent benefit. The uva ursi and bismuth also have been tried. In the case related, this plan of treatment, to a certain extent, was followed, but with how little success the result shews; indeed, when frequently occurring, or when long established, this disease appears incurable, and palliative, and not active, remedies must be employed; such a deep-rooted foundation does there appear laid for it in the broken-down, debilitated habits in which it occurs."

This paper is creditable to Mr. Anderson, and the subject is of the deepest importance to the medical practitioner.

---

### North London Hospital.

### Employment of Kreosote for the Prevention of Sickness.

The employment of specifics is tolerably ancient; the disposition to believe in them is as prevalent as ancient. But some gentlemen have a larger swallow than others. Like the ostrich, they eat all and digest all, for every fresh stone is swallowed as readily as its predecessor was. We might exhibit a catalogue of twenty drugs or more, that, within the last two or three years, have successively appeared on the medical horizon, and been hailed as a wonder-working remedy. Whatever the medicine, it always effects miracles on its first appearance; yet, oddly enough, in the course of a few months, we find it laid by, and its virtues inherited by some successor, soon to experience a similar oblivion.

Kreosote is now the rage. We dare say it is an excellent remedy; but we dare say, also, that two years hence its name will scarcely be breathed. We shall not enumerate all its valuable qualities here, but, imitating Mr. John Taylor, the apothecary of the North London Hospital, we shall simply chronicle its powers of arresting sickness.

Mr. Taylor has published nineteen cases,* in which kreosote was given to allay sickness. We shall mention the affection for which it was exhibited in each.

*Case* 1.—Vomiting from colica pictonum.
*Case* 2.—Vomiting after living in a newly-painted room.
*Case* 3.—Vomiting and severe spasmodic pain in the lower part of the abdomen, accompanied with constipation.
*Case* 4—Constipation. Vomiting after croton oil.
*Case* 5.—Vomiting and colicky pain in the abdomen.
*Case* 6.—Vomiting after colica pictonum.
*Case* 7.—Vomiting, with hysteria.
*Case* 8.—Ditto ditto.
*Case* 9.—Vomiting, with dropsy.
*Case* 10.—Vomiting, with a disease that nobody understood.
*Case* 11.—Vomiting, with the diarrhœa of phthisis.
*Case* 12.—Vomiting, with incipient phthisis.
*Case* 13.—Ascites and anasarca.
*Case* 14.—Inflammatory neuralgia.
*Case* 15.—Dropsy.

---

* Lancet, Aug. 15th, 1835.

*Case* 16.—Universal dropsy.

*Case* 17.—Aneurism of the abdominal aorta.

*Case* 18.—Hypertrophy of the heart, with dropsy.

*Case* 19.—Inflammatory anasarca.

In eighteen out of these nineteen cases the kreosote succeeded. The diseases present a copious category. The cautions to be regarded in the exhibition of the remedy are thus given by Mr. Taylor;—first, it should never be administered when there is any gastric inflammation. Secondly, the dose should be carefully regulated. We should begin with one or two drops, dissolved in an oz. of distilled water, by the aid of a little mucilage. Thirdly, thrice daily is usually a sufficiently frequent repetition of the remedy, though sometimes it ought to be given at short intervals, to keep the stomach completely under its influence.

We hope, (we can scarcely add that we expect) that the favourable anticipations of Mr. Taylor will be perfectly fulfilled.

---

### Clinical Report on Diabetes.*

Dr. Carbutt, in the volume which has already supplied some matter for the Clinical department of this Journal, has made some remarks upon diabetes, and related some cases of that obstinate disease, which are not unworthy of attention.

After noticing the various names which it has received, and the discovery, by Dr. Willis, of the presence of sugar in diabetic urine, Dr. Carbutt enquires whether such a malady as idiopathic diabetes insipidus exists.

"The question now presents itself. Are there two species of diabetes, the *mellitus* and the *insipidus*, or is there only one, the *mellitus?* Dr. Young and Dr. Mason Good consider the *diabetes insipidus* as altogether a different disease; and so it is in one of its symptoms, the absence of honey or sugar from the urine, but in every other symptom the two diseases so nearly resemble each other that they may perhaps not improperly, be considered as two species of one genus, or two varieties of one species. In speaking of John Wright's case, I mentioned that Cullen had expressed great doubts as to the existence of *diabetes insipidus* as an idiopathic disease, but that he says, 'among many cases of *diabetes mellitus*, he had observed one of *diabetes insipidus.*' Now, as for the existence of *diabetes insipidus*, as, properly speaking, an idiopathic disease, it is what I cannot take upon me to pronounce. I believe it is always symptomatic of *gastro-enteritis*, or of *gastro-entero-colitis*. Whether *diabetes mellitus* be not equally the result of gastro-enteritis, I cannot positively undertake to declare. I have not yet made a sufficient number of observations; but my belief certainly leans that way."

Thus we see that Dr. Carbutt supposes that gastro-enteritis is the cause of diabetes. The exact quo modo of this causation we shall presently perceive.

The urine of diabetes mellitus and of diabetes insipidus differs vastly in specific gravity. In the former this is very high—in the latter, it is low. Distilled water being taken at a specific gravity of 1000; the urine of *diabetes insipidus* is from 1001 to 1006: natural healthy urine is, according to circumstances, from 1005 to 1020: the urine of eleven male patients not affected with either dropsy or diabetes was taken at five o'clock in the morning, and mixed in equal quantities; the specific gravity was found to be 1017: the urine of seven female patients not affected with either dropsy or diabetes was taken at the same hour and mixed in the same manner; the specific gravity was found to be 1015. The specific gravity of the urine of the gentleman who took the above, and who laboured under no disease, was found to be at the same hour in the morning, 1013: the urine of *diabetes mellitus* is from 1025 to 1050. The sugar which the last kind of urine contains is from a tenth to a seventh part in weight.

---

* Dr. Carbutt's Clinical Reports, 8vo.

The origin of this vast amount of sugar occupies the attention of the lecturer. He shews that the vegetable substances, sugar, honey, manna, starch, and gum are identical not only in the nature, but even in the proportions of their component parts, not only in their qualitative, but in their quantitative analysis also. Mr. Donovan, Professor of Chemistry to the Society of Apothecaries in Ireland, has given an analysis of starch and sugar, computed at the mean of the results obtained separately by Berzelius and MM. Gay-Lussac and Thenard. The two analyses of starch and their mean is thus set down :—

| | Berzelius. | Gay-Lusac & Thenard. | | Mean. |
|---|---|---|---|---|
| Carbon... | 43·481.. | 43·55.. | qu. pr. | 43·51 |
| Oxygen .. | 49·455.. | 49·68.. | ,, ,, | 49·57 |
| Hydrogen, | 7·064.. | 6·77.. | ,, ,, | 6·92 |
| | 100· | 100· | | 100· |

The same operations upon sugar supply results astonishingly similar.

| | Berzelius. | Gay-Lussac & Thenard. | | Mean. |
|---|---|---|---|---|
| Carbon .. | 44·200.. | 42·47.. | qu. pr. | 43·34 |
| Oxygen.. | 49·015.. | 50·63.. | ,, ,, | 49·82 |
| Hydrogen | 6·785.. | 6·90.. | ,, ,, | 6·84 |
| | 100· | 100· | | 100· |

On these analyses, Mr. Donovan observes :—

"The result of these statements is, that starch and sugar are composed of precisely the same ingredients; that the only discoverable difference is a slight disagreement in the relative quantities, and that this is exceedingly trivial. By comparison of the two means, the following are the differences: one hundred grains of sugar contain about one seventh of a grain less carbon, about three eighths of a grain more of oxygen, and two ninths of a grain more of hydrogen than are contained in one hundred grains of starch. These are trifling differences; and, without reference to atomic considerations, it will immediately strike the inquirer that differences by far greater than these frequently occur in the analyses of the same body executed by different chemists, or by the same chemist at different times; and, in illustration, I adduce the analyses of sugar by the chemists already quoted, wherein the quantity of carbon as stated by Berzelius, is very nearly two grains more than what is stated by Gay-Lussac and Thenard. In short, we may conclude that analysis has not been hitherto able to detect any difference of composition between starch and sugar; and we may admit that, in both, the ingredients are the same in *quality* and *quantity*.

'A person who contrasts their strikingly different properties; who considers that starch is one of the most insoluble bodies, at least in cold water, and sugar the most soluble; that sugar is the sweetest of all substances, and starch the most tasteless; will naturally inquire how are these facts to be reconciled; and if the composition is the same in both substances, why are not the substances identical?

'The question is natural: at least, it would have been natural and necessary some time ago, when it was supposed that similarity of ingredients and of proportions should produce similarity of qualities. Modern discoveries have proved that this is a mistake. It is now known that, beside quality and quantity of ingredients, the peculiar mode of combination of them is to be taken into account; and, although we know, in fact, nothing about the modes of combination in which bodies exist, yet chemists have been, in a manner, compelled into this mode of explanation by the impossibility of explaining it otherwise in the present state of knowledge. In the case of starch and sugar, therefore, we know that the ingredients are the same; we may infer that the relative quantities of them are also the same; but, to assign a reason for the difference of properties, we say that they are differently combined, without pretending to say whether the difference is a closer approximation of particles, so as to expose them more effectually to each other's modifying powers; or whether it depends on some other cause. Considerations of relative specific gravities give us no information on the subject.

'Be this as it may, one would be induced to conclude that similarity of composition would give origin to a great facility of converting one sub-stance into the other; and this is just what we find to be the case in practice.'"

Gum arabic analysed by Gay-Lussac and Thenard, and by Berzelius was found, according to Dr. Henry, to consist of

| | | |
|---|---|---|
| Carbon...... | 42,23.... | 41,906 |
| Oxygen ...... | 50,84.... | 51,306 |
| Hydrogen ... | 6,93.... | 6,788 |
| | 100. | 100."* |

So that we see, to use the words of Mr. Brande and to fall back on the preceding tables for confirmation of their truth, the components of sugar differ little in their relative proportions from those of gum and starch, while the latter substance is convertible both into sugar and gum. Dr. Daubeny asserts farther, that manna enjoys a similar composition, and we are thus brought back to the original position that sugar, honey, manna, starch and gum arabic are identical, or nearly identical in the quality and quantity of their component parts. Gay-Lussac and Thenard lay down three laws, as expressions of the principal facts on this head.

"If the oxygen and hydrogen exist in the exact proportion necessary to form water, the vegetable substance is neither acid nor resinous, but it is sugar, manna, honey, starch, gum, lignin, or some such body.

If the oxygen be to the hydrogen in a proportion greater than is necessary to form water, the substance is always oil, resin, wax, alcohol, ether, &c."

Thus, as Dr. Carbutt remarks, sugar, starch, gum, &c. may be considered as composed of carbon and water only; vegetable acids may be considered as composed of carbon, water, and oxygen; wax, oils, resins, alcohol, ether, &c., may be considered as constituted of carbon, water, and hydrogen.

*" Dr. Henry's Elements of Experimental Chemistry. 7th Edition, 1815. Vol. 2nd. P. 188."

It was supposed by some that sugar was formed in the digestive organs, and might be detected in the blood. But Dr. Wollaston found that the serum of the blood does not contain a thirtieth part of the sugar which is found in an equal quantity of urine. Vauquelin and Segalas found that there was not an atom of sugar in the blood of a diabetic patient whose urine contained one-seventh part of sugar. Mr. Halliday, ingenious chemist, and teacher of chemistry at the Mechanics' Institution, examined, for Dr. Carbutt, the serum of the blood of a patient labouring under diabetes mellitus, and found that it presented no indication of the presence of either starch or sugar.

We just hinted at Dr. Carbutt's theory of the cause of diabetes; we may now present it in a well digested form. We have seen the identical, or nearly identical composition of starch, serum, and sugar, substances which form the bulk of the vegetable food we eat.—Their convertibility is well-established, and this convertibility renders it in the opinion of our author, not at all improbable that, from an imperfect or a deficient assimilation having taken place in the stomach, the gum and starch are carried unchanged to the kidneys, and are there, by a morbid process, converted into sugar.

"I consider then," he proceeds, "that sugar is formed in the kidneys of patients labouring under diabetes mellitus; and that its formation depends upon some slight change produced upon the starch and gum which are carried in an un-assimilated state to the kidneys. Now, although the detection of starch or vegetable mucilage in the serum of the blood would fully confirm the theory just laid down, yet its non-detection would be very far indeed from overthrowing the aforesaid theory. It has long been an opinion among the best physiologists, that there is a direct passage from the stomach to the kidneys; and it is a remarkable fact that substances which have not been formed in the kidneys have been detected in the urine, and not in the blood. For instance, I gave to William Grimshaw, a diabetic patient of mine, three grains of ferro-cyanate of potash, every hour for about twelve doses. I then took a portion of his urine and added to it a few drops of nitric acid; I then poured in a small quantity of a solution of sulphate of iron. The consequence was the production of a beautiful blue colour. Then abstracting about four ounces of blood, I made upon the serum a similar experiment to the one just related, without the production of any blue tint whatever: but upon adding a little of the solution of ferro-cyanate of potash, a vivid blue tint was produced. I immediately repeated the experiment upon his urine, and with the same success as before."

There can be, we apprehend, no question that, in some way or other, the sugar must come from the blood. The kidney is an excreting organ—that is clear enough. It is pretty clear also, that the blood is the pabulum of the urinary excretion. A short cut from the stomach to the kidney has never been shewn, though frequently conjectured, and not being discoverable, cannot be fairly or philosophically assumed. So far as evidence and probabilities go, the venous system is the channel of communication, and the general circulation must consequently be involved. It is therefore singular, supposing Dr. Carbutt's theory to be correct, that if gum and starch conveyed unassimilated from the stomach, be the substances from which the diabetic sugar is produced, those substances should not be distinguishable in the blood. Certainly the experiment mentioned by Dr. Carbutt appears to render this singular circumstance an argument of inconsiderable force, and we remain in a state of uncertainty and confusion. Yet if carbon and water be the elements of sugar, those elements are always existent in the blood, whether starch or gum be conveyed immediately from the stomach or not.

There is this objection to the idea that the sugar of diabetes arises from substances of vegetable origin, that such sugar continues to be voided by patients who have long submitted to an absolute restriction to the use of animal food. It is true that, in some cases, the prohibition of vegetable aliment appears to have effected the cure of the disease, but it is true also, that in the vast majority of cases in which this plan has been enforced, it has completely failed.

We have yet the pathology of the complaint to settle.

"Upon opening the bodies of those who have died of diabetes there have been found, on the one hand, all the marks of a chronic gastro-enteritis, on the other hand, a complete hypertrophy of the kidneys."

Dr. Carbutt should have added a third hand to the pathological symbol—on the other hand, he should have said, there has been found—nothing at all. We have examined some cases of the disease, and in two or three instances we really could discover no lesion on which even a sanguine pathologist could lay his finger. Our author, however, is rather confident. He continues—

"With regard to the proximate cause, or the essence of diabetes, I am disposed to agree with M. Dezeimeris,* and the more so, as I had already delivered the same opinion to you in this room, in the case of John Wright, before I had even heard of the opinion of M. Dezeimeris. The opinion is this, that the proximate or essential cause of diabetes is an irritation of the kidneys. This irritation is seldom primitive; it is most commonly only a consequence of gastro-enteritis, more especially chronic gastro-enteritis. In the course of this last mentioned disease, there comes on an excessive thirst; the patient drinks a great deal; he voids urine in proportion. This state becomes chronic. The activity of the kidneys is increased at the expense of the other excretory organs; and thus the kidneys throw out from the animal economy a quantity of fluid which must be incessantly renewed: hereby the thirst is again increased, and a circle of diseased action, thirst and diuresis, or rather hyperuresis, is established and maintained, even after the gastro-enteritis with which the disease commenced has been removed.

* Mémoires de la Société medicale d'emulation. Tome 9e.

It has been objected to this theory, that on the opening of some patients who have died of diabetes, no marks of gastro-enteritis have been discovered; and also that many patients labour under gastro-enteritis, without having shewn any symptoms of diabetes.

To these objections I have to answer; that comparatively few instances of *post-mortem* examinations of persons who have died of diabetes have taken place; that, until lately, it has not been much the custom to examine the mucous membrane of the alimentary canal, after death, in any disease, and still less in diabetes, in which disease no injury of the mucous membrane of the alimentary canal was suspected; that, in those cases in which the alimentary canal was examined, and found comparatively healthy, if any such have been, the disease might have begun with a true gastro-enteritis, which, having established the circle of thirst and hyperuresis, might subside from the operation of the vast quantity of diluting fluids taken in, and leave the thirst and hyperuresis to go on with their former energy. Lastly, to the objection that many patients labour under gastro-enteritis without having shewn any symptoms of diabetes, I can only conjecture that this may occur from the circumstance that the kidneys are not in every case alike disposed to take upon themselves the action of hypertrophy; but why they are not in every case alike disposed to take upon themselves this action is, I fancy, among those secrets of nature which we shall never be able to develope. We know not, I say in reference to this last objection, why sometimes gastro-enteritis produces headache, sometimes synochal fever, sometimes vomiting, sometimes purging, sometimes costiveness, sometimes cholera, sometimes phthisis, sometimes diabetes, sometimes both phthisis and diabetes. These are things which it is our business to study, and, if possible, discover."

We will not say that we feel very certain of the soundness of Dr. Carbutt's opinions, with respect to the existence of gastro-enteritis in diabetes. We certainly have witnessed cases of the malady, in which, so far as our memory serves us, no symptom of such a complication were presented. But examination of the bodies of the dead is the only way to decide the question. Such examination ought to be made, and to be made with care.

We agree with Dr. Carbutt, in his estimation of the prognosis of the disease—it is certainly bad upon the whole. In the majority of cases, a cure is *not* effected.

Dr. Carbutt runs over the various plans that have, at various times, been proposed or reported to cure diabetes. Dr. Rollo's animal-food diet holds, of course, the first place. It has had a long run.

"It has been reported, it really is not worth while to say where, that two cases of diabetes mellitus were cured by calcined magnesia, the one in a week, the other in a fortnight.

It is also reported, and it is equally of little importance to say where, that the diabetes mellitus has been cured by the phosphate of soda; in two cases likewise.

The celebrated Pinel, in his Nosographie Philosophique, says, 'Dans un cas de diabête causé par des chagrins profonds, et parvenu déjà au dernier degré, un malade â qui je donnais des soins l'annee passée a été gueri en sejournant à la campagne, en se livrant à un exercice régulier, en sortant de son abattement, et en insistant autant sur le régime végétal que sur toute autre substance.' "*

Opium next passes in review. Our author holds it cheap, for he believes he does not go beyond the truth when he says that, in his part of the world at least, the opium usually cures the complaint by killing the patient.

Bleeding was recommended by Dr. Watt, of Glasgow, and has been had recourse to by others. Dr. Carbutt considers this the most rational plan of all. He supposes some one to ask him his own mode of treatment. He is diffident in disclosing it, but it is this.

"Now, in the first place, if the pa-

* Pinel: Nosographie Philosophique. Tome 2nd. Paragr. ccxix.

tient's strength will admit of it, I would draw blood from the arm; I would apply leeches to the epigastrium; I would apply cupping-glasses over the kidneys; I would order farinaceous and mucilaginous diet, with barley-water, rice-water, or milk and water, to drink; I would give the patient the hydrargyrum cum cretâ, and the pulvis ipecacuanhæ compositus, in large doses; I would place the patient in a vapour bath, a sulphur bath, or a hot bath, or both, every day; I would order flannel to be worn next the skin; I would keep the patient, for the most part, in bed. If the bowels were costive, I would open them by means of castor oil. If diarrhœa existed, I would apply leeches to the belly, or to the anus, and give laudanum in chalk-mixture."

But Dr. Carbutt candidly owns his small anticipations of success in any case, and his absolute scepticism of the possibility of a cure, when the disorganization of the stomach or of the kidneys has arrived at a certain extent.

On the whole, this is a candid and a sensible clinical discourse. When such are delivered at provincial hospitals, it augurs well for the state and for the prospects of medical science in this country.

We may now take a glance at the reported cases. They are twelve in number, three being instances of diabetes insipidus, and the remaining nine examples of the diabetes mellitus. We will first present an instance of the latter, cured—or, at least, benefited so as to be considered cured.

*Case.* Francis Isdale, a farmer, married, aged 51 years, says the first accession of his disorder took place twelve months ago. He was then much exposed to the vicissitudes of the weather, and frequently got wet feet. He lived on very low diet, and suffered under depression of spirits. The first symptoms were intolerable thirst, much sweating at night, urine copious, very dry tongue, nauseous taste in his mouth, appetite pretty good, bowels costive, only one motion in two or three days, much flatus in the bowels, restless nights disturbed by dreams, occasional pain in the pit of the stomach, with a gnawing and sense of sinking before meals, pain in the right side and shoulder increased on inspiration, bilious tinge of the skin and conjunctiva, urine high-coloured, and feces clay-coloured, pain in the region of the kidneys.

All these symptoms subsided, some months ago, after three weeks' medical treatment, by Mr. Edward Lacy, surgeon in King-street, and he was well until five weeks ago, when he became affected in a similar manner. *His father died of this complaint.*

On his admission, the tongue was moist, much loaded with a white fur, the papillæ raised. He has a very nauseous taste, as of rotten eggs, in his mouth. His saliva tastes sweet. He has extreme thirst, which is best assuaged by cold water. Appetite is not so good. No vomiting or pain at the stomach. Bowels have hitherto been costive, but to-day he had two loose motions. He is much troubled with flatus. Pain on pressure in the right hypochondrium and occasionally in the shoulder. Obtuse pain in the region of the kidneys, increased by pressure. No pain in the limbs. Spirits depressed. Pulse 68, feeble. Urine is very copious, and has a sweet taste. One pint of it produced five drachms and two scruples of saccharine extract. It contains no urea."

The treatment adopted by Dr. Carbutt was confinement to bed—light diet—vapour or sulphur-baths every night—the abstraction, at four times, of fifty-six ounces of blood from the arm—five applications of leeches to the epigastrium—purgation—and the exhibition of ten grains of Dover's powder four times daily. In two months from the time of his admission, he was dismissed cured.

A table is appended, for the purpose of exhibiting the weekly weight of the patient, the daily quantity of fluid that he drank, the daily quantity of urine that he voided, and its specific gravity from day to day. The following circumstances will shew the great alteration that occurred.

On the 18th of September, the weight of the patient was 200½ ℔s; he drank

18 pints—he voided, of urine, 20 pints—and its specific gravity was 1025. On the 17th of October, he weighed 190lbs.—drank five pints—voided, of urine, 4 pints—and its specific gravity was 1011. Two or three months after quitting the hospital, the patient still passed only four pints of urine in the day.

Other cases were not so successful. The next case we shall select, for the purpose of exhibiting the results of an examination after death.

*Case.*—Oct. 10th, 1833. Henrietta Wilson, in the 40th year of her age, a soldier's wife, says she has been ill nine months. The disease came on with intense thirst, the urine greatly increased in quantity, frequent vomiting, much languor and debility, purging, appetite remarkably keen. The languor and debility have increased, so that she has been lately quite unfit to do any thing. Says her habits have always been regular and temperate, and she knows of no cause to which she can attribute her complaint, except the injury her bowels sustained from her having taken violent purgative medicines, to which she had accustomed herself every spring and fall of the year.

On her admission, she had the tongue thickly coated, particularly at the root and in the centre, with a brownish fur; it was at the tip and edges very red. She has a very nauseous taste in her mouth. Her saliva tastes sweet. The skin is dry and rough, never moist. Appetite is at present less keen than before. She feels very sick and faint, and a sinking in her stomach, as if she were going to die. She does not vomit now. She has an intolerable thirst, and makes an excessive quantity of urine, which, she says, has a sweet taste. She has a troublesome cough at night, and expectorates a quantity of very nauseous matter. Some pain on the left side of her chest on respiration and on coughing. Considerable pain on pressure over the epigastrium and right hypochondrium. Says she cannot lie comfortably on the left side. Sleep is much disturbed by terrifying dreams, as of danger of falling from some precipice, or of being drowned. Bowels at present are costive, but they always have been loose on her taking opening physic: says she feels best when her bowels are confined. Complains of much feebleness and languor, occasional pain in her limbs, as the calves of her legs, knees, arms, &c. Pulse 112, very small and feeble. One pint of her urine contains, of saccharine extract, 370 grains. It apparently contains no urea.

We need not particularise the treatment, as the patient sank under gastric and pulmonary symptoms, and died on the 18th.

*Autopsy, 22 Hours after Death.* The mucous coat of the stomach was inflamed at various points. The mucous coat of the duodenum, jejunum, ileum, and colon was also strongly marked by inflammation, as well as discoloured in many parts. There were no appearances of ulceration, except something like incipient ulcers in the duodenum.

The right kidney was considerably enlarged, or, as it may be termed, *hypertrophised.* The left kidney was nearly without substance, and presented an appearance of complete *atrophy.* The ureters of both sides were preternaturally enlarged.

The uterus was tuberculated. The liver presented an unhealthy mottled appearance.

On cutting into the chest, there were strong marks of pleuritis on the left side, such as adhesions of the pleura, with lymph and pus observed on the surface of the lung. The upper lobe of the left lung contained a very large vomica; the posterior portion was hepatised. No apparent disease in the right cavity.

One of the cases was that of a boy, six years and three months old. The child only weighed 35½ pounds, and he passed daily above 27 pounds of urine, one pint of which was found to contain 624 grains of saccharine extract. An extraordinary case! As may readily be supposed, the result of the treatment was not fortunate.

In the instance of a factory youth, aged 17, affected with the diabetes insipidus, the sulphur and vapour-bath, with the compound powder of ipecacuan, leeches to the stomach, and castor oil when necessary, proved quite successful.

The only other case which we shall notice is that of a labourer aged 64, affected with the diabetes insipidus. On admission the quantity of urine voided daily was nine pints, of the sp. gr. of 1006 to water at 1000. He had passed as much as twenty-eight or thirty in the twenty-four hours. The patient died, and for pathology's sake we allude to the case.

"Autopsy, twenty-four hours after death.

3d. Upon opening the abdomen, the stomach externally presented, near the cardiac orifice, the appearance of perforation of its coats. Internally, the mucous membrane seemed much inflamed, and there was a large ulcer which penetrated through all the coats. The inner coat also presented several small ulcers. The parietes of the stomach were much thinner than natural. The pylorus was very thick and inflamed.

In the duodenum was a larger ulcer, with several smaller ones.

There were numerous ulcers throughout the small intestines, but they were particularly obvious and large in the latter portion of the ileum, where it terminates in the caput cœcum coli; in this spot they were very numerous. The large intestines were also much inflamed and ulcerated. In the lower part of the rectum the mucous membrane was much thickened, and had the appearance of dark inflammation. Ulcers were also observed in this portion.

The kidneys seemed diseased, particularly the left one, which was larger than natural, much congested, and had a mottled, granulated appearance internally, with something like cysts, which might be enlarged infundibula.

There was a scrofulous-looking abscess under the left pectoralis muscle, not communicating with the interior of the thorax. The second rib, upon which this abscess was situated, was carious.

There were numerous and strong adhesions of the pleuræ on both sides. The lungs, when cut into, appeared healthy.

The liver appeared healthy."

We think this lecture will repay the reader. It presents a very fair and judicious account of a disease, which, unfortunately, we do not understand, and of treatment which, unhappily, is usually unavailing.

---

### St. Marylebone Infirmary.

We observe that Dr. Clendinning, the able and zealous physician to the Marylebone Infirmary, has published a statistical report of the diseases observed in that institution between the 22d of May and the 24th of July, 1835. We think we need not remind our readers of our anxious and repeated efforts to induce the medical officers of charitable institutions to publish clinical reports. We have been amongst the first, if not the very first—we certainly have been the most persevering in making this call upon our brethren. Some have and some have not responded to it. We have little doubt that before a very long period elapses, a systematic series of clinical reports will be presented. The signs of the times in this respect are evident. For our own parts, we shall forward, as far as we are able, the attempts of those who display so highly praiseworthy a spirit as Dr. Clendinning, of the Marylebone Infirmary, and Dr. Macleod, of St. George's Hospital.

We would suggest the combination of statistical reports, that is, summaries of facts, and individual cases. For the purposes of general utility, we should say that statistic records, and collections of cases calculated to display the general laws of disease or treatment, are preferable to insulated instances of rare complaints. The latter are too often chosen, because they excite curiosity and interest. The accomplished surgeon or physician is too apt to measure the attainments, the appetites, and wants of the mass of the profession by his own. Things familiar to him he too readily concludes to be equally familiar to all, and hence the prevalence of transcendental pathological papers in our journals and transactions. One sound and universal induction is worth much more, in an useful point of view, than the most extraordinary fact, or the most imposing theory. Such inductions should be the aim of those who write for the real instruction of the public—of our clinical reporters. It is with facts that they have essentially to do, and their object should be, to transfer the experience of the ward to the report.

Dr. Clendinning apologises for the present imperfect condition of his Tables. He regrets that he has not yet been able to procure sufficient materials for columns indicating ages, trades, birthplace, and so forth—all circumstances of interest, and some of consequence. We shall content ourselves with extracting the Table supplied by the worthy Doctor to the Medical Gazette, referring the reader for remarks on individual subjects to the pages of our contemporary.

" *Table of the Diseases of Marylebone, as observed in the In and Out-door Practice of the St. Marylebone Infirmary, from Friday, May* 22, *to July* 24, 1835.

| | Male. | Female. |
|---|---|---|
| *Fevers.* | | |
| Gastro-enterite .. .. | 1 | 5 |
| Variola .. .. .. | 2 | 3 |
| Rubeola .. .. .. | 14 | 8 |
| Scarlatina .. .. .. | 3 | 1 |
| Febris infant. remit .. | 1 | 0 |
| Febris.. .. .. .. | 30 | 30 |
| *Inflammations.* | | |
| Cynanche .. .. .. | 2 | 2 |
| Catarrhus .. .. .. | 1 | 0 |
| Pneumonia.. .. .. | 7 | 7 |
| Iritis .. .. .. .. | 0 | 1 |
| Hydrocephalus.. .. | 1 | 1 |
| Phlegmon .. .. .. | 1 | 0 |
| Bronchitis, chron. .. | 0 | 1 |
| Hepatitis .. .. .. | 0 | 1 |
| Meningitis.. .. .. | 0 | 1 |
| Dysenteria.. .. .. | 0 | 1 |
| Bubo .. .. .. .. | 0 | 1 |
| Encephalitis .. .. | 2 | 2 |
| Gangrena penis.. .. | 1 | 0 |
| Ophthalmia .. .. | 25 | 2 |
| Inflam. pedis .. .. | 1 | 1 |
| Febris puerperalis .. | 0 | 1 |
| Bronchitis .. .. .. | 9 | 9 |
| Erysipelas .. .. .. | 0 | 1 |
| Hordeolum .. .. | 0 | 1 |
| Erythema .. .. .. | 0 | 1 |
| Pleuritis .. .. .. | 3 | 7 |
| Peritonitis .. .. .. | 2 | 1 |
| Rheumatismus .. .. | 17 | 17 |
| Laryngitis . .. .. | 0 | 1 |
| Otitis .. .. .. .. | 0 | 1 |
| Anthrax .. .. .. | 1 | 0 |
| Inflam. bursæ .. .. | 0 | 2 |
| Gastritis .. .. .. | 0 | 1 |
| Abscessus .. .. .. | 3 | 7 |
| Hernia humoralis .. | 1 | 0 |
| Tumor faciei .. .. | 1 | 0 |
| *Chronic Organic Diseases.* | | |
| Phthisis .. .. .. | 30 | 20 |
| Carcinoma.. .. .. | 2 | 0 |
| Tabes.. .. .. .. | 1 | 0 |
| Morb. chron. cerebri.. | 1 | 1 |
| Morb. cordis .. .. | 2 | 1 |
| Morb. chron. ventric.. | 2 | 0 |
| Morb. chron. cordis.. | 5 | 3 |
| *Diseases of Nervous System.* | | |
| Apoplexia .. .. .. | 3 | 8 |
| Neuralgia .. .. .. | 0 | 1 |
| Delirium tremens .. | 1 | 0 |
| Mania .. .. .. | 11 | 8 |
| Cephalalgia .. .. | 0 | 1 |
| Pertussis .. .. .. | 3 | 1 |
| Ramolissement .. .. | 0 | 1 |
| Epilepsia .. .. .. | 2 | 1 |
| Paralysis .. .. .. | 1 | 1 |
| *Discharges.* | | |
| Hæmoptysis .. .. | 2 | 2 |
| Hæmorrhag. uteri .. | 0 | 2 |
| Diarrhœa .. .. .. | 2 | 1 |
| Amenorrhœa .. .. | 0 | 2 |
| Menorrhagia .. .. | 0 | 1 |
| Hematemesis .. .. | 0 | 2 |
| Leucorrhœa .. .. | 0 | 1 |
| *Cutaneous Affections.* | | |
| Psora .. .. .. .. | 4 | 20 |
| Strophulus.. .. .. | 1 | 0 |
| Eczema .. . .. | 0 | 1 |
| Eruptio syphilitica .. | 0 | 1 |

| | Male. | Female. | | Male. | Female. |
|---|---|---|---|---|---|
| Urticaria .. .. .. .. | 2 | 0 | Atrophia .. .. .. .. | 2 | 0 |
| Porrigo .. .. .. .. | 10 | 10 | Prolapsus ani .. .. | 1 | 1 |
| | | | Fractura costæ, &c. .. | 2 | 0 |
| *Miscellaneous.* | | | Syphilis .. .. .. | 2 | 5 |
| Fistula in ano .. .. | 1 | 0 | Ustio .. .. .. .. .. | 0 | 1 |
| Vulnus .. .. .. .. | 2 | 0 | Syncope .. .. .. | 1 | 0 |
| Ulcus .. .. .. .. .. | 9 | 9 | Hydrops .. .. .. | 0 | 3 |
| Asthenia .. .. .. .. | 0 | 1 | Fistula lacrymalis .. | 0 | 2 |
| Contusio .. .. .. .. | 3 | 4 | Ebrietus .. .. .. | 1 | 1 |
| Hernia .. .. .. .. | 2 | 1 | Morb. spinæ .. .. | 0 | 1 |
| Icterus .. .. .. .. | 1 | 0 | Podagra .. .. .. | 1 | 1 |
| Concussio cerebri .. | 1 | 0 | Anasarca .. .. .. | 0 | 2 |
| Pyrosis .. .. .. .. | 0 | 1 | Aphthæ .. .. .. .. | 0 | 1 |
| Sequelæ partus .. .. | 0 | 20 | Obstipatio .. .. .. | 0 | 2 |
| Abortus .. .. .. .. | 0 | 6 | Luxat. humer. .. .. | 1 | 1 |
| Dyspepsia .. .. .. | 1 | 3 | Senectus .. .. . | 2 | 1 |
| Tympanitis .. .. | 0 | 2 | Retentio placentæ .. | 0 | 1 |
| Incerta .. .. .. .. | 7 | 7 | Luxatio .. .. .. | 1 | 0 |
| Colica pictonum .. | 2 | 0 | Aneurism poplit. .. | 0 | 0 |

*Table of Deaths occurring in the Wards of the St. Marylebone Infirmary, from May 22d, to July, 24, 1835.*

| | Male. | Female. | | Male. | Female. |
|---|---|---|---|---|---|
| Consumption .. .. | 11 | 6 | Convulsions .. .. | 1 | 2 |
| Inflammation of lungs, | 2 | 2 | Atrophy, and .. .. } | 1 | 1 |
| Concussion of brain .. | 1 | 0 | Marasmus mesenteric. } | 0 | 1 |
| Inflammation of brain, | 2 | 2 | Epilepsy .. .. .. | 0 | 1 |
| Cancer of liver .. .. | 1 | 0 | Chronic dysentery .. | 0 | 3 |
| ——— womb .. | 0 | 1 | Aneurism of aorta .. | 0 | 1 |
| ——— breast .. | 0 | 1 | Diseased brain .. .. | 0 | 2 |
| Apoplexy .. . .. | 1 | 2 | Typhus .. .. .. | 0 | 1 |
| Croup.. .. .. .. | 0 | 1 | Puerperal fever .. .. | 0 | 1 |
| Ulcer of stomach .. | 0 | 1 | Old age .. .. .. | 0 | 1 |
| Diseased heart .. .. | 0 | 3 | Dropsy .. .. .. | 2 | 1 |
| Asthma .. .. .. | 0 | 3 | Diseased stomach .. | 1 | 0" |

The number of deaths is greater on the female than the male side. It is much to be regretted that Dr. Clendinning has not obtained the ages of the patients, and that he has not distinguished the in and out patients of the institution. He promises future reports, and we hope he will be enabled to render these and other matters more distinct.

---

### Clinical Report on Hernia.*

Dr. Macfarlane relates nine cases of hernia in that volume of clinical facts, from which we have already freely culled cases which must ever be valuable, whatever theories prevail. We think we discover an increasing disposition on the part of hospital surgeons and physicians to render their stores of useful knowledge available to the mass of their professional brethren. This disposition we would anxiously encourage, and we have done our best at all times to promote it.

Case 1. *Strangulated Scrotal Hernia—Intestine burst by the Taxis—Operation—Death.*

J. P. æt. 39, admitted Feb. 19, 1827, at 7, p. m. He had been subject for twenty years to a reducible inguinal rup-

* Clinical Reports of the Glasgow Infirmary. By J. Macfarlane, M.D. 8vo.

ture of the left side; and, during the last two years, a small portion of it remained irreducible, and prevented him from wearing a truss. The symptoms of strangulation had existed for ten hours; and, during a considerable part of that time, a surgeon had made powerful and continued efforts to return the displaced parts. These produced at first a good deal of pain in the tumor; which, however, along with the vomiting, ceased for several hours, after he felt as if something had been pushed into the abdomen.

One of Dr. Macfarlane's colleagues employed the taxis rather forcibly for about ten minutes ineffectually. Cold was then applied to the tumor, and a colocynth enema exhibited. In about an hour after this, he was seen by Dr. Macfarlane.

The tumour was then about the size of the fist,—had a pyriform shape, an irregular surface, and was rather firm and doughy anteriorly, but smooth and elastic posteriorly,—the outer part being apparently omentum, behind which a fold of intestine was probably situated. The lower part of the scrotum was as large as a child's head, the swelling being chiefly confined to the left side. It was tense, smooth, and of a livid colour. The integuments were considerably thickened; and they crackled under the fingers, when firm pressure was applied, similar to what is observed from the effusion of fibrine around a sprained joint. There was also obscure fluctuation, apparently from a collection of fluid in the cavity of the scrotum. These appearances evidently depended on the effusion of blood, both into the integuments and the interior of the scrotum, which was to be attributed to the forcible pressure employed previous to his admission. His pulse was 100 in the minute, the respiration slightly hurried, and the bowels obstructed; but there was no anxiety of countenance, and only slight pain in the belly; the vomiting had ceased, and, altogether, the symptoms were so mild that an operation was not immediately called for; yet it will be afterwards seen, that before this period the injury was done to the bowel which led to a fatal result.

Dr. Macfarlane considered it improper to reapply the taxis, in consequence of the previous attempts, and of the swelling of the scrotum. At 1, a. m., the patient was much worse. No alvine evacuation had been procured; the abdominal pain was acute and diffused; he vomited every ten minutes; his pulse was 140, small and sharp; his breathing hurried, and his countenance sunk and anxious. The scrotum was now as large as a man's head, and nearly its whole surface was purple and discoloured.

At 4, p. m. the operation was performed. When the sac was opened, fully a pound and a half of dark-coloured blood escaped; a considerable quantity of which was pressed up from the depending part of the scrotum. The hernial tumor consisted of a large piece of omentum, which was covered with coagulated blood, and of nearly two feet of intestine. The omentum was contused and lacerated, and the protruded gut, which was afterwards found to be the ileon, was almost wholly separated from the mesentery. It contained several rents, which passed in a longitudinal direction; and into each of these openings two or three fingers could be introduced. There had been no escape of feculent matter, but the gut was flaccid, and of a deep purple colour; the discolouration depending not on gangrene, but on the extravasation of blood into the cavity of the bowel, and between its coats.

" Two strictures, about an inch separate, one at the outer, and the other at the inner ring were divided. I then attempted to return the uninjured parts of the bowel into the cavity of the abdomen; but after several attempts this was found impracticable. The impediment did not arise from the smallness of the hernial aperture, or from the presence of adhesions; but in consequence of the empty and relaxed state of the bowel, and the great distention of the parts within the abdomen, I found that although a small portion was pushed up before the finger, it was impossible to prevent it from being instantly reprotruded.* From the dreadful condition of the parts in this unfortunate patient, I had no alternative, but either to allow the bowel to remain in the sac, covering it with a poultice or other emollient application, or to excise it. Had the patient survived the effects of the disease and operation, I have no doubt, from the great injury which the bowel had sustained, and from the extensive destruction of its mesenteric attachments, that it would soon have become gangrenous, which termination would probably have been accelerated by the constant irritation to which so large a piece of intestine must have been exposed in an open wound. The only prospect of benefit from treatment, seemed to consist in procuring, as speedily as possible, a free feculent discharge, through an artificial anus; and as this could be effectually obtained only by dividing the gut as it emerged from the inguinal canal, I proceeded to excise the bowel; an operation, it must be acknowledged, of the most formidable kind, and only warrantable under the desperate circumstances I have attempted to describe.

From the vascular state of the small portion of the mesentery attached to the bowel, I found it necessary to pass a ligature around it to prevent hemorrhage; the gut was then divided in two places and removed. Both ends of the bowel were secured in the wound; they did not collapse, but retained their natural calibre from thickening of the coats by previous inflammation. A piece of omentum, nearly the size of the fist, was also removed; after which two stitches were inserted into the lower half of the wound, and simple dressing applied."

The patient was much exhausted after the operation, the vomiting was incessant, the abdomen became tympanitic, and in seven hours he died.

As Dr. Macfarlane justly observes, it is impossible to furnish a more useful or impressive commentary on the danger of employing force in the reduction of a hernia than by the case now detailed. The disease was of twenty years'standing; the intestine was free, although the omentum was adhering to the sac; the inguinal ring was large; and every thing was in the most favourable state for the successful use of the taxis; nevertheless, it was productive, and that in the hands of a well-informed surgeon, of the most disastrous consequences; the omentum, intestine, and mesentery were torn, and an immense effusion of blood produced.

Even within these last few years, a sensible alteration has occurred in the practice of English surgeons. The taxis is now much less employed, and the operation is sooner had recourse to. Within the sphere of our own observation, the results have been gratifying in a high degree. The mortality of strangulated hernia is diminished, patients are spared many hours of barbarous, because unnecessary, surgical interference. Even so lately as ten years ago, a patient brought into a large London hospital, with strangulated hernia, was tolerably sure of undergoing a lengthened application of the taxis at the hands of the house-surgeon, of the surgeon to whom his case belonged, and of several, if not all the surgeon's colleagues. The taxis was used before the warm bath, in, and after it, and, if any thing approaching to a glimmer of hope appeared to remain, the taxis, after an interval, was again employed, perhaps with cold, perhaps as a pendant to a tobacco-glyster. The fatality of such cases was much greater than it now is. At present, a moderate employment of the taxis, if ineffectual, and, generally speaking, a trial of it in

* "Boyer gives a case which occurred to Petit. Although the stricture was divided, and the gut found to be free of adhesions, it could not be returned. He was advised to cut off the part, but he allowed it to remain in the wound, and covered it with pledgets of linen; the greater part of it returned spontaneously into the abdomen, the wound healed, and a cure was accomplished."—*Traité des Malad. Chir.* *tom.* viii. *p.* 126.

the warm-bath, is usually succeeded at once by the performance of the operation. That operation is, *per se*, attended with so little danger, that its risk scarcely deserves consideration, and, altogether, we may look on its present period and mode of performance as a great improvement in the treatment of the disease.

There are some, even at present, who cling to the lengthened use of the taxis. They reason from a few cases. No doubt the operation, being early performed, is performed in some cases where it might, by patience and delay, be avoided. But the general results must determine the general value of a mode of treatment, and those general results are undoubtedly in favour of the early operation. If there are cases in which it may be dispensed with, those are exceptions, and must be received as such. The rational mode of giving an exception its proper weight is, not by rejecting the general rule, but by endeavouring strictly to appreciate the circumstances under which exceptions occur, and by recognizing them when they appear. In the instance of hernia, we are not to return to late operations, because early ones are now and then unnecessary; but we are carefully to study the symptoms, and observe the course of individual cases, and arrive, if we can arrive, at the power of distinguishing those, in whose favour the general principle of treatment may be waived.

It is obvious that general rules, in medicine, are necessarily rules of principle—their application constitutes the sum of the experience and the judgment of the surgeon or physician. In the case before us, the principle is this:—that an early operation is safer and more successful than a later one. In applying that principle, it is equally clear that, cæteris paribus, the greater the information of the surgeon the more likely he is to apply it aright—the less his information, the more likely he is to apply it awrong. The extension of the argument implies that, if the surgeon's knowledge be perfect, his application of the principle will be also perfect. Unfortunately, however, human knowledge is not perfect, and we cannot expect that the application of any principle, especially in medicine, where the disturbing circumstances are so numerous, shall be free from liability to error. It follows that, to diminish that liability, the way is, not to reject an useful rule, but to endeavour to ascertain the cases to which it does and does not apply. But to proceed.

Dr. Macfarlane observes that, in old herniæ, the gut sometimes becomes diseased, and, whether attenuated or thickened, it is generally softened. The taxis, in such cases, is particularly hazardous. As an instance of this, he relates the following case.

"In May, 1827, when walking along one of the public streets of this city, I was requested by a surgeon to accompany him into his shop, and examine a poor woman who had fainted a few minutes after the reduction of a strangulated crural hernia on the right side, which had annoyed her for more than twenty years. The tumour had been in a state of strangulation for about eight hours; but the symptoms were not urgent. After a gentle use of the taxis for five minutes, the hernia went up with the usual noise, and was almost immediately followed by syncope. On examination, I found her skin covered with cold perspiration; the pulse rapid and feeble; and she complained of acute pain in the right inguinal region. She was immediately carried home, but continued to get worse. The pain became diffused; vomiting and hiccup occurred; the belly was tympanitic; and, without re-action taking place, she died in less than seven hours. I was present at the dissection, and found a lacerated opening in the middle of the ileon, which could admit three fingers. The intestine was softened and thickened, but not in a state of gangrene; and through this preternatural opening, the contents of the alimentary canal had escaped and excited peritonitis."

It is difficult, indeed it is impossible, to say what amount of force should be ventured on in the application of the taxis. Much must depend on the history of the case, the state of the parts and the condition of the patient, and all must be determined by the judgment of the surgeon. It must certainly be better to do too little than too much.

Passing over a case in which the patient refused, for some time, to submit to the operation, and in which the application of cold was followed by erysipelas, we may stop to notice a case, in which a portion of the omentum was excised.

Case.—*Strangulated Entero-Epiplocele—Excision of a Portion of the Omentum—Cure.*

Mrs. M. æt. 58, had laboured under a strangulated crural hernia of the right side for forty-eight hours, on the 10th of December, 1826, when Dr. Macfarlane saw her. The tumor, about the size of half an orange, elastic and painful; the abdomen was tumid, tender on pressure, and the seat of frequent paroxysms of spasmodic pain; the countenance was flushed; the pulse 60 and sharp. After trying the taxis, &c. Dr. Macfarlane proceeded to perform the operation. The sac contained thickened omentum, and a small fold of dark-coloured intestine. The stricture was divided, and the intestine was easily returned; but, as the protruded omentum was diseased, it was cut off, the adhesions it had contracted to the neck of the sac were destroyed, and it was then replaced in the abdomen. The sac was greatly thickened, and so separated from the surrounding parts, probably by the efforts made to reduce the tumor, that Dr. Macfarlane thought proper to excise it. The patient had acute peritonitis, requiring copious bleeding, leeches to the abdomen, &c. and two days elapsed before the bowels were freely evacuated. After this, the unfavourable symptoms disappeared, the wound healed by the first intention, and in three weeks she was able to wear a truss.

The objections to excision of the omentum are, the liability to subsequent bleeding. The abdominal portion of the cut omentum may retract into the abdominal cavity, and furnish there a troublesome, or even serious hemorrhage. If the vessels are tied, and the ends of the ligature cut short, the threads may give rise to peritoneal inflammation or to abscess. A case of this sort occurred to Mr. Earle. He excised a portion of omentum, and, having tied the vessels on the cut surface of the remainder, removed the ends of the ligatures. Some time afterwards, an abscess formed at the umbilicus, and the ilgature-knots were discharged. The best plan, when it is necessary to remove a part of the omentum, is to excise it, and to leave one end of the ligature long, as is done on stumps or other wounds. The knot can then be removed in due time. Some have recommended tying the portion of omentum, and allowing it to slough away. We have seen this done, and succeeded by abscess in the sac, and extensive suppuration in the neighbourhood.

The next case is curious on several accounts.

Case.—*Strangulated Entero-Epiplocele—Operation—Existence of a Mesenteric Hernia—Death.*

R. M. æt. 28, admitted Jan. 4th, 1827. The left side of the scrotum was occupied by a pyriform tumor; from the upper part of which, a hard, irregular, well-defined cord, about two inches in length, was felt passing up, and entering the inguinal ring; it had more the feel of a thickened and enlarged cord, than of the neck of a hernial tumor. On grasping and raising up this band, however, a more elastic part was felt below, where an impetus was recognized on coughing; and here the pain, on pressure, was intolerable. The tumor fluctuated distinctly at the anterior, inferior part of the scrotum; but the greater part of it was hard and irregular, and could not be distinguished from the testicle. Twelve years previously, a hernia had suddenly appeared in the left groin. It was easily reduced, and kept so by a truss, until five days prior to his admission. It then descended, whilst making a strong effort, and had resisted all attempts at its reduction. Dr. Macfarlane concluded that the cord-like substance, so distinctly felt, was thickened omentum, behind which was probably a strangulated fold of intestine. He urged an operation, but the patient refused to submit to it.

The symptoms grew worse, and had the characters that usually wait on strangulated intestine, in an advanced stage. When we mention stercoraceous vomiting, hiccup, tympanitis, and a pulse of 130, the rest may probably be guessed. On the evening of the 6th, he at last consented to the operation, and it was performed. The sac contained one ounce of dark-coloured, rather fetid fluid, and a large, livid, soft mass of omentum, apparently gangrenous, which was intimately adhering to a fold of intestine, three inches in length, lying at the posterior part. The gut, which was of a deep chocolate colour, but not actually gangrenous, had an opaque spot on its surface, apparently from a deposition of lymph. About one-half of the omentum was removed, the other half adhered to the bowel so intimately, as to render its excision impracticable. The stricture was divided, the adhesions of the omentum and intestine to the neck of the sac were destroyed by the finger, and the parts returned into the abdomen.

The symptoms were not materially relieved, and, at 9, p.m. of the 9th, he died, seventy hours after the operation.

*Inspection.* The small intestines were enormously distended, of a dark red colour, adhering to each other by recent lymph, and to the abdominal integuments on the left side of the pubes. The omentum, which was tightly stretched over the intestines, and attached to the portion of gut which had been strangulated, was dark-coloured, and so soft as to yield readily to the fingers. There was no effusion into the abdomen, but the intestines were smeared in some places with brownish pus. The ileon was found to have passed deeply into the pelvis, through an opening in the mesentery. It had not contracted any preternatural adhesions, but it was evidently constricted and gangrenous, and was with difficulty drawn out from its unnatural position. The upper part of the same bowel had been included in the inguinal hernia; it was doubled on itself, adhering, and in a state of gangrene.

The next case presents nothing of interest or consequence. The following is more worthy of attention. Before we enter on it, we are under the necessity of dissenting from a remark of Dr. Macfarlane's. He observes that it is but rarely that the omentum enters into the formation of a crural hernia. This does not agree with our own experience. It is true, that omentum is more frequently included in an inguinal than in a femoral hernia; but still it is far from uncommon in the latter. We remember many cases in which we have seen it.

Case.—*Strangulated Crural Entero-Epiplocele—Intestine included in an additional Sac of the Omentum.*

"I was requested (says our author) by Mr. William Easton, surgeon in Calton, to visit Mrs. C. aged fifty-five, at nine o'clock, p.m. on the 31st Jan. 1832. She had been labouring for thirteen hours under the symptoms of a strangulated hernia. The tumour, which occupied the situation of the right crural opening, was about the size of a small hen's egg; it was tense, painful, and turned up over the outer surface of Poupart's ligament; there was also swelling, tension, and pain of the right side of the abdomen; vomiting and constipation. The pulse was slightly accelerated: the countenance anxious; and there was a good deal of prostration of strength. She had had a reducible crural hernia of the left side for many years, but had never observed a tumour in the right groin till that morning, when it suddenly protruded, and became strangulated.

Having failed by a moderate use of the taxis, I immediately proceeded to the operation. On opening the sac, a small quantity of limpid serum escaped, and a considerable portion of omentum presented itself. This was not in its usual soft doughy state, but was of a globular shape, and had a tense elastic feel. On minute examination it was found, that either fluid, or probably a portion of intestine, was contained in a sac formed by the omentum. I therefore pinched it up with a pair of forceps, and made a cautious opening into it, when a considerable quantity of serum escaped: it was then laid open to the same extent as the peritoneal sac, and found to inclose a knuckle of dark-coloured intestine. The stricture was divided; the gut returned; the protruded omentum, which had contracted slight adhesions to the neck of the sac, was cut off; the bleeding, from one of its vessels, was arrested by torsion; the adhesions were destroyed, and the rest of it returned: immediately after which more than an ounce of limpid serum escaped from the cavity of the abdomen. She speedily recovered."

It is not very uncommon to find the intestine involved, more or less, or concealed by omentum. We have seen several instances of this description, and, occasionally, some embarrassment in consequence. A recollection of their possibility is always proper, and frequently necessary, on the surgeon's part.

In the next case brought forward by Dr. Macfarlane, no fluid existed in the sac; on dividing the stricture, which was very tight, more than an ounce of straw-coloured fluid escaped from the abdomen. The Doctor observes, and very truly, that if there is little or no fluid in the sac, it is occasionally difficult to ascertain when the latter is really laid open. He has twice seen the fascia propria mistaken for the sac, and the gut returned into the abdomen with the sac unopened. Death was the result. It is generally better, when any doubt exists, to satisfy ourselves that the gut is really exposed, and the stricture divided, by drawing down the former in a slight degree. The great point, however, is to be sure of the division of the stricture, for the cases related by Mr. Key are sufficient to shew that, if that division has been properly effected, the sac may often be safely returned into the abdomen.

The next case presents no feature of consequence. The last is an example of the cure of artificial anus. With this we shall, therefore conclude the present series.

Case. R. C. æt. 60, admitted Nov. 11th, 1831. He had had reducible scrotal hernia of the left side for thirty years, and of the right for twelve; there had also been, on the latter side, a hydrocele for more than a year. This had been punctured about a fortnight before his admission, and the operation had given rise to inflammation and gangrene of the scrotum. The sloughing was very extensive on his admission. On the 22d, Dr. Macfarlane found, on removing a loose portion of the slough, which evidently involved the septum scroti, that a small livid opening, on the inner side of the left hernial sac, was exposed, through which there was a copious discharge of thin feculent matter. It was now evident that the gangrene had extended to the intestine, and that an artificial anus was formed. Through this opening, the whole of his stools were passed for nearly three weeks, when it completely closed, and the alvine matter resumed its natural course. The scrotum assumed a healthy granulating appearance, and had cicatrized fully two-thirds, when the symptoms of diseased heart, under which he laboured, became more urgent, and general dropsy took place. He died on the 5th Jan. following.

On examination after death, the inguinal openings, on both sides, readily admitted four fingers. Both sacs contained considerable portions of the small intestines, which were free of adhesions, and appeared to be healthy. The part of the gut in the left hernial tumor, in which the artificial anus had existed, could scarcely be distinguished. All that was discoverable was, that a small portion, about the size of a sixpence, was slightly thickened, irregular, and purple-coloured.

As Dr. Macfarlane observes, the peculiarity, in this instance, of cure of artificial anus, is the circumstance, that no adhesion of the bowel to the neighbouring parts took place.

### St. George's Hospital.

### Report of Cases treated from January 1st to July 1st, 1835. By R. Macleod, M.D. &c.*

Dr. Macleod introduces his report with the following observations.

"It is frequently urged as matter of reproach, against the physicians and surgeons of our hospitals, that they publish no reports of their practice; and some have not scrupled to assign motives for this silence far from creditable to the parties in question. I believe the true cause is to be found in that which I know has influenced myself;—I allude to the disinclination which men naturally feel to claim attention without having something which they deem of importance to communicate. My reason for stepping out of the beaten path, and following the example recently set in your journal by one of the physicians to the St. Marylebone Infirmary, is not from conceiving that I have any thing so valuable to offer, that the withholding it would be an *irreparable* injury to the public, but because I think the complaint above alluded to is not without some justice, and because I am of opinion that accurate returns of the number of patients labouring under particular diseases, and of the results—even did the reports contain nothing more—would soon become valuable in a statistical point of view."

We agree with Dr. Macleod in thinking, that the accusations of a desire to monopolize knowledge, not unfrequently brought against hospital surgeons and physicians, are absurd. The number who can be influenced by such a feeling must be small indeed. But Dr. Macleod takes no notice of a motive, which is probably the most influential of all—we allude to indolence. Four out of five of the medical officers of our public institutions do not publish reports of their practice or their cases, simply because it is not an integral part of their duties, and it is not volunteered. We trust that the example of Dr. Macleod, an active and able hospital physician, will stimulate others, who, perhaps, are not convinced of the necessity of exertion, to contribute their share to the advancement of the knowledge of particular facts, or statistical results. We congratulate the physicians of St. George's Hospital, on the opportunities afforded to them in its spacious wards, and we are sure the public will obtain much valuable information from that source.

The number of beds occupied in the hospital is now 300. The applications being much more numerous than the vacancies for applicants, the worst cases are, in consequence, selected. This renders the ratio of mortality high, but affords proportionate facilities for the cultivation of pathological anatomy. In fact, the examinations of the dead are conducted so carefully and systematically at this hospital, that probably the pupil enjoys more advantages, in this respect, at St. George's, than at any other institution. This, however, by the way. We shall merely notice the statistic portion of Dr. Macleod's communication.

"On the 1st of January, 1835 (he says), there were in the hospital under my care 30 patients. There have been admitted, 97. Total, 127.

| | |
|---|---|
| Of these, there have been disposed of | 101 |
| Remained under treatment, July 1 | 26 |
| Total | 127 |

Those 'disposed of' are as follow; viz:—

| | |
|---|---|
| Discharged cured | 59 |
| ——— relieved | 17 |
| ——— complaints stationary | 8 |
| Transferred to Surgeon | 3 |
| Dead | 14 |
| Total | 101 |

The 14 deaths were produced by the following diseases:—

* Med. Gaz. July 25, 1835.

| | |
|---|---|
| Phthisis, common | 2 |
| ———— affecting chiefly the larynx | 1 |
| Suppuration of thoracic glands, | 1 |
| Tuberculated peritoneum, with ascites | 1 |
| Malignant disease of stomach | 1 |
| Tuberculated liver, with ascites, | 3 |
| Encephaloid disease of liver | 1 |
| Organic disease of the heart | 3 |
| Apoplexy | 1 |
| | — |
| | 14" |

We are next presented with a list of the cases of affection of the head, the respiratory organs, the heart, the fauces, and the abdominal viscera.

The diseases of the cerebral and nervous system are :—

Apoplexy, 2.—Cured, 1 ; dead, 1.
Epilepsy, 1.—Relieved, 1.
Hemiplegia, 1.—Relieved, 1.

SPINAL DISEASE.

Paraplegia, 1.—Under treatment, 1.

FUNCTIONAL NERVOUS DISEASES.

Hysteria, 2.—Cured, 2.

The diseases of the respiratory organs are :—

Laryngitis, acute, 1.—Cured, 1.

Do. chronic, 1.—Cured, 1.

Do. phthisical, 3.—Relieved, 1 ; dead, 1 ; under treatment, 1.

Bronchitis, acute, 3.—Cured, 1 ; dead, 2.

Do. chronic, 1.—Cured, 1.

Pulmonitis, 3.—Cured, 3.

Phthisis, 4.—Relieved, 2 ; dead, 2.

Suppuration of the bronchial glands, 1. —Dead, 1.

The diseases of the heart are :—

Acute pericarditis, 1.—Cured, 1.
Chronic disease of the heart, 14.—Relieved, 8 ; dead, 3 ; under treatment, 3.

DISEASES OF FAUCES.

Cynanche tonsillaris, 1.—Cured, 1.
Syphilitic ulceration of the fauces, with eruptions, 2.—Cured, 2.

DISEASES OF ABDOMINAL VISCERA.

Peritoneum, tuberculated, with dropsy, 1.—Dead, 1.
Do. chronic inflammation of, 1.—Cured, 1.
Stomach, anomalous *gurgling* of, 1.—Stationary, 1.
Do. scirrhous ulceration of, 1.—Dead, 1.
Do. supposed malignant disease of, 1. —Stationary, 1.*
Bowels, obstipation of, 1.—Cured, 1.
Do. colica pictonum, 1.—Discharged for irregularity, 1.
Do. diarrhœa, 1.—Cured, 1.
Do. dysentery, 1.—Under treatment, 1.
Do. ulceration of (?) after fever, 1.—Cured, 1.
Liver, chronic disease of (tubercular), with ascites, 6.—Relieved, 2 ; under treatment, 1 ; dead, 3.
Do. encephaloid disease of, without ascites, 1.—Dead, 1.
Do. chronic inflammation of, 4.—Cured, 3 ; under treatment, 1.
Do. biliary obstruction—jaundice, 1.—Cured, 1.
Spleen, chronic enlargement of, 1.—Stationary, 1.
Abdominal tumors, nature not ascertained, 2.—Under treatment, 2.

The treatment of fever, so far as the cases go, is satisfactory in an eminent degree.

Fever, common continued, 6.—Cured, 6.
Do. intermittent—quotidian, 1.—Cured, 1.
Do. do. irregular, 1.—Cured, 1.

Dr. Macleod speaks highly of mercury, in the management of continued fever. He thinks that that medicine, exhibited at the commencement of the disease, is as much a specific as bark in ague. In every one of the above instances, when the gums became affected, the febrile symptoms ceased.

Dr. Macleod relates the particulars of several interesting cases, in addition to the preceding statistical reports. For these, however, we must refer to our contemporary.

---

* Since dead. The patient left the hospital, to avoid having his body examined after death.

### Royal Infirmary of Edinburgh.

### Clinical Report for the Winter Session of 1834-35. By J. Syme, Esq. Surgeon to the Royal Infirmary.

Mr. Syme, whose name we need scarcely introduce to our readers, has not merely commenced, (for he did that long ago,) but systematically pursues the plan of presenting clinical reports. We have little doubt that, ere long, we shall have a considerable body of valuable facts periodically and regularly presented to the public. This is as it should be. The objects of medical institutions are, after all, the diffusion of useful medical knowledge, and that diffusion is strictly synonymous with the general diffusion of medical facts.

The present report extends to the length of twenty-seven pages in our old and valued Northern Contemporary. We can only, at present, select a few of the cases for notice.

*Medullary Disease of the Eyeball.*

Mr. Syme relates two cases, and we notice them in this brief paragraph for one purpose, and one only—to express a desire that the operation of excision of the eye-ball for medullary disease should be entirely abandoned. The operation is, we may almost say with perfect truth, invariably unsuccessful, and the sooner it is given up the better.

*Injury of the Head—Secondary Symptoms—Matter under as well as over the Dura Mater.*

We draw attention to this case, because it illustrates an important circumstance, the occurrence of matter beneath the dura mater, opposite the spot where it is found external to it.

James Swinton, aged 34, hackney-coachman, was admitted on the 7th of January, soon after being found lying insensible by the policeman on duty at the Calton Hill. It was ascertained that he had gone up to look for a London steam-boat which was expected, and finding that it had arrived, hastened down to get his coach ready for the passengers, when, the rocks being slippery from frost, he had fallen about 16 feet, and lighted with his head on the stone steps at the foot. When brought to the hospital he was very restless and incoherent, as if intoxicated, which, however, he was not, though reported to be of irregular habits. There was a very large wound of the scalp, extending from the root of the nose to the vertex, the edges of which were very uneven and much lacerated. The bone was laid bare to the extent of several inches in length. Soon after admission he became composed and sensible. In the evening the pulse was 84, but the skin was hot, and he complained of great pain in the head. ℥xxvi. of blood were taken from the arm, and gr. v. of calomel, with xv. of *Pulv. Jalap.* given as a purgative.

Next day, though he was much better, it seemed proper to repeat the bleeding to the extent of ℥xvi.

On the third day his pulse was 104, and he complained much of headach. The tartrate of antimony was ordered in nauseating doses.

On the fourth day he was delirious and violent, so as to require the strait jacket. ℥xx. of blood were taken.

On the fifth day he had not slept, and was very restless.

On the sixth day he was very noisy and unruly; passed his stools in bed, and seemed in a hopeless state.

On the seventh day he was more composed, so that the jacket was removed. He had slept a little towards the morning, pulse 96.

During the six following days he went on most favourably. He was sensible and cheerful. The sloughs separated from the scalp; great part of the exposed bone granulated; the pulse, tongue, and appetite were natural.

On the 14th day a great change had taken place. He complained of more pain in the head; the tongue was dry and brown; skin hot; pulse 100. He was bled to the extent of ℥x. with considerable relief; and resumed the solution of tartrate of antimony.

During the three succeeding days he had frequent severe rigors, and the wound assumed a dry glassy appearance. As there could be no doubt that suppuration had now taken place within the cranium, while the integrity of the patient's sensorial and voluntary powers seemed to show, that the brain and its immediately investing membranes were not seriously injured, it was thought right to perforate the bone. This was done on the 17th day by means of the *trepan,* which was set on the bare portion of the skull, about the anterior extremity of the sagittal suture. The bone was quite dead and perfectly dry throughout its whole thickness.—So soon as it was divided, a quantity of thin, extremely fetid pus, by computation at least ℥ss. gushed out.

During the three following days he had frequent rigors, and was occasionally incoherent. The pulse was very variable, ranging from 84 to 130. But his appearance was on the whole not unfavourable; he was quite sensible, and the wound looked well.

On the 21st day he became worse, passing his stools in bed; swallowing and articulating with difficulty; and suffering from convulsive twitching of the muscles, with hiccup. He died on the 22d day.

On dissection, the dead portion of the bone was found defined by an ulcerated groove on the outer as well as inner surface of the skull. The *dura mater* was covered with puriform lymph for some distance beyond the dead portion of bone, and at one part was so soft and thin, that it scarcely bore the pressure of the forceps. Under the *dura mater,* and nearly to the same extent as the superjacent effusion, there was a similar deposition in the subarachnoid cellular texture. The other contents of the cranium were found in their usual condition."

This case is well detailed, but we think that the examination of the body after death was incomplete. We need not tell even our *un*surgical readers now, that abscesses in the liver and the lungs are common in connexion with secondary suppuration within the cranium. The symptoms are the same, but the knowledge of the combination is important, because it leads us to expect less from an operation, and to look with more suspicion upon symptoms, than otherwise we might be led to do. We recollect some well-marked cases of this description. A man had an injury of the head, which was succeeded by symptoms of suppuration on the dura mater. He was trepanned, and the matter was disclosed, if not discharged. But rigors continued, and he died. On examination, matter was found beneath the dura mater, and deposites of matter existed in the lungs.

On the whole, we must look on the operation of trephining for suppuration within the cranium as, generally speaking, excessively unfortunate. We never *saw* a case in which it was successful. Mr. Pott relates a large number, but no one is now-a-days so lucky as was Mr. Pott. Mr. Syme's experience, we see, coincides with our remark.

This case, says he, has been selected from many very severe and some fatal injuries of the head, because it was the only one that has occurred for a long while, in which the internal suppuration was so seated and limited, as to afford any promise of advantage from the operation of *trepan.* And, had it not been for the unfortunate morbid action under the *dura mater,* there can be little doubt that the patient would have recovered.

In the latter supposition we know not if we can entirely agree with Mr. Syme. But that is too uncertain to be argued on.

The following case is interesting, as exhibiting the state of a fracture two months after the occurrence of the accident.

" *Simple Fracture of the Os Femoris—Reunion—Death at the end of Two Months—Dissection.* Susan Barr, aged 51, was admitted on the 2d of April in consequence of having sustained a fracture of the left thigh-bone, which she stated had happened the preceding evening from being thrown down by a man who ran against her while crossing the street. The injury having been ascertained to be seated in the upper third of the bone, the limb, properly supported by splints, was placed upon a double-inclined plane.

On the 15th she was suddenly seized with sickness and vomiting, and then became extremely hot and restless, with dry brown tongue and quick pulse. In three or four days these unpleasant symptoms left her, and on the 20th of May the limb was found sufficiently firm to be freed from restraint. On the 27th she had a rigor and a return of her former symptoms, which continued with progressive aggravation until the 7th of June, when she died.

The fracture had evidently been comminuted. The broken surfaces remained *quite unconnected,* a soft bloody semi-fluid substance only lying between them. In the medullary canal there had been a deposition of osseous matter in a sort of granular state, and the external edges of the fracture were united by bridges of dense bone. In this case, then, the *provisional* callus of the French pathologists was nearly completed.

It is a remarkable fact in the history of pathology, that Duhamel's theory of the reunion of fractures, which was founded on an erroneous analogy between the formation of wood and that of bone, has proved to be much nearer the truth than that of Haller and his pupils, who entertained correct opinions as to the formation and nourishment of bone. Duhamel supposed, that, in a case of fracture, the periosteum had its inner layer converted into bone, just as the inner layer of the bark of a graft is converted into wood, and that thus a connecting bridge was formed between the broken bones. When specimens were shown to him of the union extending through the medullary canal, he explained the appearance by alleging, that the *internal* periosteum had suffered a similar change; and when his attention was called to sections of old united fractures, in which a compact mass of bone occupied the seat of the fracture, he was satisfied with supposing, that the external and internal periosteum had united. Rude and crude, and ill-founded as this theory was, it approaches wonderfully near the enlightened views of Breschet and Dupuytren, who have been the first to explain satisfactorily the process by which the every-day accident of fracture is repaired. The reader is no doubt aware that the explanation formerly admitted, of an organizable substance effused from the broken bones into the space between them, and gradually hardened into bone, is quite untenable; and that the process of reunion truly consists, 1. in the formation of a capsule surrounding the fractured extremities by thickening and condensation of the neighbouring tissues; 2. the deposition of bone in this capsule, and in the medullary canal; 3. the growth of bone from the surrounding osseous surfaces until the cavity is completely obliterated. The second stage is generally so far completed in from three to six weeks, that the limb regains its rigidity sufficiently to resist any moderate force, and the cure is then said to be completed; but the real cure requires at least as many months. The case that has just been related affords a striking illustration and confirmation of this process; since, if it were not for the provisional callus or bridges of new bone connecting the external edges of the fracture, the bone would still be flexible, and, in fact, one of the halves is flexible from the section having been accidentally made so as to leave the bridge more on one side than the other."

With this case our now narrow limits compel us to conclude. We recommend the whole report to the philosophic lovers of facts.

---

### SIR P. DUN'S AND MERCER'S HOSPITALS.

Dr. J. Osborne has lately published a paper on diseases of the stomach, in our Dublin Contemporary, from which we shall extract a few cases.

CASE 1. *Gastrodynia.*

" Ellen Maguire, aged 21, admitted into hospital, October 3d. *Pain in the region of the stomach, generally when it is empty; the pain is relieved by eating; sour eructations, and vomiting of sour fluid, attended with relief of the pain;* catamenia absent four months; meat agrees best with her stomach; pulse natural. Those symptoms have lasted nearly three years.

Camphorated senna mixture ad effectum.

℞. Nitrat. argenti, grana iv.
Aquæ distillat. ℥viij.
M. Sumt. ℥ss. omni hora.

7th. At first some nausea was experienced after taking the solution, which has ceased. The pain is diminished, and the vomiting has never occurred since.

10th. Bowels torpid, otherwise free from complaint.

℞. Mass. pil. galbani. comp. ʒi.

Extract. aloes aquos. ʒss.

M. Ft. pil. xxiv. Sumt. iij. omni nocte.

Pain and vomiting not having recurred, she was dismissed in a few days."

Case 2. *Gastrodynia—Scirrhus.*

"Mr. P., aged 39, came under my care, 24th February, 1833, with a pain in the epigastrium, not increased by pressure. Vomiting of watery acid fluid, to which he has been subject nearly nine years; *the vomiting eases and sometimes entirely removes the pain;* appetite good until within the last few days; food now mostly rejected; pulse seventy-four; tongue white, but moist; abdomen feels empty, and rather retracted; bowels habitually costive, has not been freed during the last three days; all the purgatives taken having been rejected by the stomach. He was placed under mercurial treatment, his mouth became sore, and a slight effect was produced on his bowels. In consultation with an eminent practitioner of this city, since dead, several efforts were made to produce copious evacuations, but in vain. The pulse became more frequent, the stomach more irritable, and at length he could retain only cold water. In compliance with the anxious desire of his friends, and with the consent of Dr. Crampton, whose assistance was now obtained, we agreed that he should make a trial of fluid mercury. He took a pound weight, being nearly an ounce by measure in my presence. He swallowed it in two deglutitions, and immediately gasped for breath in a state of great anxiety, then vomited, but none of the mercury came up. No effect on the bowels was produced, and in the vomiting which continued during the following days, the mercury appeared in minute globules, having caused no appreciable inconvenience while in the stomach. The matter vomited was on different occasions bloody, and of a fetid odour. During the last four days he would submit to no further remedies; all having proved ineffectual, either to cause action of the bowels, or ease from the vomiting. He gradually sank, and died on the 13th of March. On examination of the body, the pyloric end of the stomach was found above half an inch in thickness, which gradually diminished towards the cardiac extremity, where it entirely disappeared. At the lesser curvature, towards the pylorus, there was an ulcer about the size of half-a-crown, with jagged edges, which had perforated through the scirrhus mass in this part, and had reached the peritoneal membrane. The pylorus was so contracted, that an ordinary-sized quill could scarcely be passed through it. The interior of the stomach, at the pyloric half, was uneven from masses of scirrhus projecting into it; but except at the ulcer, the mucous membrane was in a state of integrity, being of a reddish purple colour. No emaciation had taken place until within the last three weeks, and, notwithstanding the enemata and other means used to move the bowels, there was a considerable quantity of fæces in the large intestines, but unaccompanied by air."

It is not a little curious that no remains of the quicksilver were found in the stomach or bowels.

Dr. O. relates some cases illustrating the insidious advances which the disease makes, "as long as the greater curvature remains in a healthy state." The following is one of these cases.

"Hugh Reilly, aged 77, died in Stevens' Hospital, in October, 1832, with aneurism of the aorta, and imperfect closure of the aortic valves, bronchitis, and general dropsy. His appetite was remarkably good up to within a few days of his death, nor had he vomiting until this period, when it was generally brought on by coughing. He did not at any time after his admission refer either to the region of the liver or stomach, as the seat of pain. On examination after death, there was found, close to the cardiac orifice of the stomach, a cancerous ulcer, about the size of half-a-crown. It was raised above the level of the mucous membrane, the edges composed of a soft fungous mass, were irregular, inverted, and everted, the centre was depressed and hard, very irregular, and of a greyish colour. An interval of only one-third of an inch separated its edge from the cardiac orifice. The mucous membrane near the pylorus, and along the lesser curvature was very rough, softened, and presented a punctated injection. The mesenteric glands were enlarged, hardened, and converted into scirrhous structure. The liver was not enlarged, but was studded throughout with scirrhous masses, varying in size from that of a pea to the largest walnut; their shape irregular, and those on the surface projecting far above it, with raised edges and depressed centres. Those were hard, white, and striated when cut into, presenting the appearance of a bad turnip. Some of them were of a creamy consistence. The gall-bladder was filled with black and very viscid bile. Kidnies quite healthy."

Several other illustrations are given, and many judicious observations are appended to them, for which we must refer to the Journal itself.

---

### Lock Hospital,

**Notice of two Cases of Syphilitic Sore in the Urethra. By Henry James Johnson, Esq.**

The following are two examples of this rare affection. I say rare affection, because, though it is probable that venereal ulcerations occur now and then in the urethra, beyond the sphere of vision, the evidence of their existence is too slight to permit much confidence to be placed in it. In the following cases that evidence was sufficient to produce conviction in my mind, and it probably will not appear altogether inconclusive to my readers.

The first case was that of an out-patient at the Lock Hospital. It is unfortunate that my notes of the case have been lost, but the following are the principal particulars.

*Case* 1. A young man, a mechanic, applied to me on account of a discharge from the urethra, pain in micturition, and painful erections at night. Without making a minute examination of the penis, a precaution, however, which, should always be adopted whenever a venereal patient presents himself, I looked upon the case as one of gonorrhœa and treated it accordingly. But it did not yield to the remedies prescribed, and it appeared peculiarly obstinate. Nearly three months had elapsed from the first appearance of the disorder, and discharge still existed, with painful erections at night. The pain was referred to a spot in the urethra, distant about an inch from the orifice. Here I could feel distinct induration. But other symptoms had now occurred, which directed my attention more particularly to the induration I have mentioned. The patient complained of sore-throat, and superficial yellowish ulceration was visible upon the tonsils. An eruption had also appeared upon the skin. It consisted of slight depositions in the cutis of a brownish red colour, desquamating freely. It was what may be called the syphilitic psoriasis. In order to ascertain as perfectly as possible the nature of the indurated spot in the urethra, which I suspected to be really a venereal ulcer, I separated the lips of the orifice as widely as possible, with a fine pair of dressing forceps, and was enabled to obtain a glimpse of the spot in question. It appeared to be distinctly ulcerated.

Under these circumstances, I did not hesitate to prescribe a course of mercury. The patient was ordered to take the blue pill, and all local applications to the urethra were abandoned. Under this plan he rapidly improved, and ultimately he perfectly recovered. The discharge soon ceased, the induration subsided, the secondary symptoms disappeared, and in short, the patient appeared completely cured. Whether a subsequent return of the secondary symptoms took place, I am unable to determine, as I have not seen the patient since the period at which he was dismissed.

*Case* 2. A gentleman consulted me about the middle of August of the present year, on account of a complaint under which he had been labouring for four or five months. He said that he had been treated for gonorrhœa.

There was profuse purulent discharge from the urethra. On the right side of the orifice of the urethra was a distinct, yellow ulceration, partly external, principally extending into the urethra. But this was not all. For two inches from the orifice the urethra felt hard, irregular, and thickened, excessively painful upon pressure, and when pressed giving exit at the orifice to an increased quantity of discharge, slightly tinged with blood. On the breast and back was an eruption of pityriasis, yet attended with rather more thickening of the cutis than is usual in that cutaneous affection. On the abdomen were several scattered spots, exactly resembling the syphilitic psoriasis. I told the gentleman I was confident he had ulceration of the throat, with such a combination of the symptoms. He said that he did not believe he had any thing the matter there. I examined the throat and found each tonsil extensively ulcerated. The ulceration was of a yellowish colour, superficial, without much surrounding redness.

The history of the case was indistinct. The patient had been living uninterruptedly with a female friend, but he had also had connexion at intervals with other women. It appeared that he had been subject for some time to ulcerations on the glans. He had had what was considered the gonorrhœa for four or five months, or more. The eruption on the skin had existed for two or three months also, but the spots of psoriasis had been of late occurrence. As he was not aware of the presence of ulceration in the throat, he was ignorant, of course, of the date of its occurrence.

Under these circumstances, I could not hesitate to believe that the patient laboured under syphilitic ulceration in the urethra. The eruption on the skin, the ulceration in the throat, the irregularity and tenderness of the urethra continuous with evident ulceration at the orifice, and finally the inutility of remedies directed for some time against the supposed gonorrhœa, appeared to form a chain of evidence strongly in favour of the syphilitic nature of the malady.

In a paper in a former number of this Journal, I directed attention to a very troublesome complication or consequence of gonorrhœa—chronic inflammation of the corpus spongiosum urethræ. In that affection the corpus spongiosum may be felt indurated, is tender to the touch, keeps up the discharge from the urethra, and sometimes gives rise to abscess in the cellular tissue of the penis. But in chronic inflammation of the corpus spongiosum, there are never, so far as I have seen, secondary affections of the throat or skin, nor is it attended with ulceration at the orifice of the urethra.

I prescribed a course of mercury for this gentleman—the application of mercurial ointment to the inferior surface of the penis—injections of dilute blackwash into the urethra—and the use of the tepid bath. At the date of this report (Sept. 1) but a short time has elapsed since this plan of treatment was commenced. Yet the ulceration at the orifice of the urethra has healed—the induration of the corpus spongiosum has diminished, the eruption is fading, and the ulceration of the throat has decreased. So far as they go, these results are favorable to the view I have taken of the case.

I have detailed these cases because they are facts which have happened under my own observation, not because they present any thing very rare or extraordinary. Other surgeons have probably met with similar cases, which the publication of these may recal to their recollection. The practical point they are intended to enforce is, the necessity of making a careful examination of the state of the urethra in all obstinate cases of urethral discharge.

---

### EDINBURGH EYE INFIRMARY.

REPORT OF THE EDINBURGH EYE INFIRMARY, FROM NOV. 20, 1834, TO MAY 25, 1835.*

This report is made by Mr. Watson, who, in January of the present year, published an abstract of the cases that occurred in the six months preceding November, 1834. In fact, the institution has only been in existence since July, 1834. The total number of cases admitted in the year has been 544. Those received between the 20th of November, 1834, and the 25th of May, 1835, have been 276. Of these, four have been admitted as in-patients; the rest were placed in the books as *out-patients,* some of whom were visited at their own dwellings. Many of the individuals were affected with diseases in both eyes, which, in some, were of a different nature; so that the number of cases of disease of the eye, admitted into the institution, was considerably greater than the number of patients above stated. The number of patients affected with each disease were as follow:

| | |
|---|---|
| Ophthalmia tarsi . . | 27 |
| Inversion of eyelids . . | 2 |
| Tumors of eyelids . . | 9 |
| Ulceration of eyelids . | 2 |
| Ulceration of eyebrow . | 1 |
| Painful nervous affection of eye, | 1 |
| Paralysis of muscles of eye, | 5 |
| Fistula Lacrymalis . . | 5 |
| Injuries of eyeball from foreign bodies . . . | 19 |
| Inflammation of conjunctiva (acute and chronic) . | 75 |
| Inflammation of conjunctiva (purulent) . . . | 8 |
| Strumous ophthalmia (chiefly pustular). . . . | 37 |
| Inflammation of cornea . | 13 |
| Inflammation of membrane of aqueous humour . . | 3 |
| Iritis . . . . . . | 9 |
| Opacities and specks of cornea, | 21 |
| Ulcers of cornea . . | 8 |
| Staphyloma of cornea . | 1 |
| Contracted and irregular pupils | 2 |
| Amaurosis . . . | 17 |
| Cataract, single and double, | 9 |
| Atrophy of eyeball . . | 2 |
| | 276 |

The next table exhibits the number of operations, and the gross results of treatment in all the cases.

| | Operations for cataract. | By extraction. | By needle. |
|---|---|---|---|
| Penman, | 1 | | 1 |
| Skinner | 1 | | 1 |
| Anderson, | 1 | 1 | |
| Brown, | 1 | 1 | |
| | — 4 | — 2 | — 2 |
| Artificial pupil | | 1 | |
| Fistula lacrymalis, | | 5 | |
| Tumours of eyelids, | | 6 | |
| Vascular specks, | | 2 | |
| Inversion of eyelids, | | 1 | |
| | | 15 | |
| Total No. of operations performed . . | | | 19 |

---

* Edinb. Medical and Surg. Journal, July, 1835.

| | | |
|---|---|---|
| No. of cases admitted . . . | | 276 |
| cured . . . | 191 | |
| relieved . . . | 23 | |
| incurable . . | 20 | |
| irregular in attendance | 6 | |
| died . . . | 1 | |
| still under treatment, | 30 | |
| operations recommended . | 5 | |
| | 276 | 276 |

To these statistical results are appended some remarks on particular points. Mr. Watson points out the marked distinction between acute and chronic conjunctivitis. In these, however, we see little novelty or peculiarity.

*Injuries of the Eye from Foreign Bodies.*

"One of the most remarkable effects of injury of the eye, is that of immediate blindness, from concussion of the nervous part of the eye. Two striking cases of this kind occurred at the Eye Infirmary, from the detonation of percussion caps, which have of late come into general use for the explosion of fire-arms. In both of these, small portions of the percussion caps had struck the eye-ball, but without seeming to have produced any very serious wound. Slight inflammation followed, but complete blindness was immediately produced, though the eye retained its natural appearance. These accidents shew the great danger of using such caps, and that more effectual means should be employed to prevent it."

It seems difficult to imagine any great amount of concussion to arise from a small portion of a percussion-cap impinging on the eye. A metallic fragment of that sort is either impelled with violence, or it is not. If it is, it must surely be driven into the textures it impinges on, that is, it must occasion more or less of a wound; if it is not impelled with such violence as to occasion this effect, we repeat that it is difficult to conceive its producing what can be considered as concussion. The facts, however, are superior to mere reasoning, and no doubt they are correctly reported by Mr. Watson.

"Another very remarkable effect of a wound of the eye-ball is atrophy of the globe. This probably takes place from the want of regeneration of the humours of the eye at the part wounded, in consequence of inflammation destroying its secreting powers. The wound may be slight, yet the same effect follows; even that produced by the introduction of a couching-needle is not unfrequently the cause of collapse or atrophy of the eye to a greater or less extent.

In one very remarkable case of collapse of the eye-ball, resulting from an injury with a piece of glass seven years previously, an attack of inflammation came on, and the eye enlarged to a much greater size than the other, with great pain from the rapid effusion of fluid. When the inflammation abated, the eye again became somewhat collapsed. The inflammation of several different parts in the interior of the eye appears to occasion dropsy or dropsical effusion in the globe. In two preparations in my possession, of dropsy of the eye-ball from internal inflammation, the dropsical fluid is exterior to the retina in one, collapsing it into a cord in the centre of the eye; in the other, the fluid is within the retina, situated between it and the hyaloid membrane of the vitreous humour, which is pressed forwards."

*Iritis.*

Our author has met with several severe cases of this disease, which resisted mere antiphlogistic treatment, but yielded satisfactorily to mercury. We conceive that, unless special circumstance forbad the exhibition of that medicine, no well-informed surgeon would think of treating a severe case of iritis without it. It would be like engaging in a bout of fisticuffs, and voluntarily tying up the right hand.

*Cataract.*

During the last six months, only four cases have been submitted to operation. In two, the cataract was extracted—in the other two, it was broken up by the needle. The following case is instructive.

"The case of the last patient, (Brown) in whom extraction was performed, was a very remarkable one, and requires particular notice. He was a glass-blower, 54 years of age, corpulent, and bloated in appearance, and was much addicted to the immoderate use of spirits. In prospect of the operation, I put him on a regimen of preparation, advising him to abstain from strong liquors, to live moderately, take exercise, and a small quantity of Epsom salts every second morning. This I found afterwards had only been partially attended to. He underwent the operation on the 18th of February, and bore it well. On the day after, the 19th, he had no pain or complaint, but from his appearance I was afraid that *delirium tremens* was coming on. I regretted I was obliged to go to a considerable distance from town, but left him to the care of the house-surgeon, Mr. J. P. Rae, and my friend Dr. Craigie, who visited him on the morning of the 21st. On the 20th, there was no pain or inflammation of eye, but slight symptoms of *delirium tremens* came on during the night. In the course of this afternoon, he became restless and unmanageable, then furiously maniacal, requiring restraint in bed. 21st, Passed a very restless night, and the scalp was hot, and covered with profuse perspiration; the pulse was from 120 to 130, and he was in a state of constant agitation, spoke incessantly, and in a state of furious delirium.—There is no inflammation of eye. Bleeding by cupping from the nape of the neck, cold applications to the head, antimony and opium were the remedies administered; but he continued to become worse, and sunk into a low state, which was followed by death about five o'clock on the evening of the same day. On *dissection*, the vessels of the brain were much injected with blood; copious sero-albuminous effusion was found beneath the arachnoid coat; and the ventricles were dilated, and contained a considerable quantity of serous fluid. The eye operated on presented a very favourable appearance.

The above proved a most unfortunate case, and had a very unexpected termination. The symptoms were those of high inflammation of the membranes of the brain,—a conclusion which was confirmed by dissection. In this case it turned out upon inquiry that the individual, who drank habitually, never to intoxication, but always to maintain himself under the influence of the unnatural *stimulus*, had laboured under repeated attacks of *delirium tremens*, with spectral illusions, and the other symptoms of deranged cerebro-meningeal circulation. Many such patients seem to become affected with the symptoms of *delirium tremens* from their wonted strong drink being suddenly withheld from them, and in other instances from its being taken in a slight extent more than usual. These cases generally prove very unmanageable, and too frequently fatal, whatever course of treatment is prescribed."

The surgeon who witnesses much hospital practice has frequent opportunities of seeing such cases as the one detailed above. Mr. Watson says, what perfectly accords with our own experience, when he observes that these cases are frequently fatal. If the reports of Baron Dupuytren and of others were to be received with implicit credit, the traumatic delirium or delirium tremens would appear a very manageable complaint. The fact, however, is, that a large proportion of those affected with it sink whatever are the means employed. We are disposed to think that a certain degree of inflammatory action of the membranes of the brain is very frequently mixed up with those symptoms which are usually considered, and which probably are of a purely nervous character. On the examination after death of persons who have sank with all the symptoms of delirium tremens, we have observed, as in the case here detailed by Mr. Watson, sero-albuminous effusion beneath the arachnoid membrane, effusion of serum within the ventricles, and vascular injection of the substance of the brain. That low inflammation should occur in such cases, and in such individuals as furnish examples of this disease, can scarcely excite any well-grounded surprise when we reflect that, under similar circumstances, and in similar constitutions, low inflammation of the cellular tissue of the surface of the body is extremely common. Neither need we be greatly surprised that treatment of all kinds is very often unavailing. It is probable that this is most unsuccessful when inflammatory action exists in addition to the purely nervous delirium. For these patients bear depletion so ill, that this, which is necessary to remove the inflammation, tends to aggravate that state of system in which the disease originated.

At the same time it is satisfactory, and in some instances may be highly useful to know, that delirium tremens is occasionally complicated or succeeded by inflammation of the arachnoid. For this the surgeon should watch, and when he has reason to believe that it exists, he should be cautious of his stimulants, and feel his way, as well as he is able, with opiates, and local depletion or blisters. It must be owned that the management of such a case will too often be unproductive of satisfactory results, however judicious it may be. But the more appropriate, the more likely it is to be successful; and if we are prevented from effecting good, we can enjoy the reflection that we have done no harm.

### The Hospitals and their Regulations.

Such is the title of a leading article in our contemporary the London Medical and Surgical Journal. Our readers will do us the justice to believe that in the observations we are about to make, we are actuated by no feeling of illiberality. Our medical politics cannot with any degree of fairness be charged with a tendency of that kind. At the present time, when our own as well as other institutions are submitting or about to submit to the alterations rendered necessary by the changes which the diffusion of education has effected in the tempers and opinions of men, it is necessary that sound and judicious principles should be inculcated in periodical publications. Enthusiasm betrays many to consider, and interest actuates some to insist that whatever is, is *not* right, and that to change every thing established is the only thorough reformation. The hospitals and all connected with them, are the objects of unceasing and unmitigated hostility, and the catalogue of the abuses perpetrated under the sacred veil of charity, is, if we may believe the representations of some writers, of the most appalling and disgusting character. There is an ancient adage that the devil is not so black as he is painted, and we verily believe that the hospitals and the hospital "functionaries," are not the incarnations of injustice they are sometimes said to be.

The writer in the London Medical Journal has the hospital-phobia in a very high degree. The symptoms are of an aggravated character. After noticing the magnificent exterior of our hospitals, and the liberal spirit which in this country prompts individuals to dedicate such noble revenues to charity, and after vouchsafing a nod of satisfaction at the cleanliness, order, and comfort seen within, he passes to the *corps d'élite*, the medical and surgical officers of the institution. The picture is now changed—the hydra-heads of corruption rise—and the Reviewer lays about him right lustily to prostrate them gory and mangled in the dust.

The reviewer supposes a spectator walking into the wards and board-room. He is a spectator of the right sort, a thoroughpaced reformer, determined to be liberally dissatisfied with every thing and every one he meets. Well then the spectator has the *corps d'élite* before him, and taking a look at the awkward squad, he finds them distributed into "head surgeons, their assistants, and apothecary, house surgeons, and dressers." He marvels that within the apparent terra firma of charity, extortion and peculation flourish abundantly.

" Such an inquirer would find, in the course of his investigation, that the head, or principal surgeons, received an enormous remuneration; not from the funds of the establishment, but in fees from students resorting to it for information. He would find the assistant-surgeons touching no reward in the aforesaid way, but waiting for their turn to do so in succession, as their Gamaliels became defunct, or retired from the field. He could not fail, nevertheless, to perceive that a certain harvest was reaped by them in the interim; owing to the prominent situations they held. The apothecary might perchance be in his shop and so escape scrutiny, but the house-surgeon would be looking sharply after some hospital assistancy, or infirmary, and paying for standing on his elevation. Lastly, the dressers would pass in review; each in his own estimation a profound 'chirurgical,' but *nevertheless* paying for his post. After these would follow a promiscuous phalanx paying for being the witnesses of the feats of all the former. All this would our inquirer behold, and he could not fail to feel surprise that, in the house of charity, *payment* in some way or another was the order of the day,—patients excepted, all parties would be fingering cash, either in receipt or disbursement.

" Looking at the amount of money collected from house surgeons, dressers, and forlorn-looking students (we speak of a large hospital), our friend would say, 'Here is money enough to support this glorious establishment extracted out of the pockets of those who avail themselves of the advantages it presents for acquiring knowledge,—doubtless the pelf is appropriated to that laudable purpose.' The rejoinder would be 'No! two or three principal 'chirurgicals' pocket all, leaving their assistants the hope of doing the same at some future period.' The inference he would draw from this exclusive dealing would be 'either these head surgeons are men of indefatigable exertion, and almost supersede the necessity of their unpaid helpmates, or they are the foremost of the eminent, a sort of magicians in their art!' What beadle or porter in a hospital would not smile at this conclusion? Not because it bears any thing extravagant in itself, but because the misapplication of the funds in question has been so long continued, that what is unjust and monopolising on the face of it, has been rendered indistinct and obscure by the rubbing of long usage.

" Disappointed at finding disinterestedness among the heads, the assistant surgeons receiving nothing, and chewing the cud of *hope*, would engage his attention, and probably his wonder at the eagerness with which men sought and contested for that office. He would say, 'here are charitable gentlemen, active and good, struggling to be employed in what brings them no remuneration—men doing good for its own sake!' But as disappointment when begun is apt to recur, so would it here. The disinterested assistant would be found rioting on loaves and fishes, which his attachment to a hospital procured him, by advancing his private practice."

The gist of all this lacrymation is, that hospital surgeons and physicians are paid. Really we see no abstract injustice in this; on the contrary, we think, and many others think so too, that the public services of medical men are not paid enough. A ruinous competition has ground down the profession into a starveling race—fellows who, like the opposition coachmen, not only take their passengers for nothing, but regale them with brandy and water on the road. Our dispensaries and many of our infirmaries have the services of physicians and surgeons for nothing — our hospitals have those services gratuitously also, so far as the hospital funds are concerned—and the remuneration, such as it is, is derived from the attendance of pupils. Now we do not see that Mr. A. or B. has any natural or indefeasible right to come from Yorkshire to London, to walk into the first hospital that presents itself, and accosting an *unpaid* physician or surgeon, to request him to shew him his practice, explain his views, and exhibit his skill in the manner best adapted for his convenience. We find no such principle laid down even in "The Rights of Man by Tom Paine," and we think it would be difficult for the most ingenious Owenite to prove it. But setting aside the claim to remuneration on the part of the medical officers of hospitals, and disregarding rights, let us look upon the question as one of expediency. In the first place, it may be stated as a general law, that men work better when paid for their exertions than when unpaid. Perhaps editors are a class of men whose liberality is as great as can be expected from any members of a civilized community. None are so eloquent as they on the necessity and advantages of liberality and disinterestedness—none can be more earnest in pointing out abuses and jobs and peculation. Yet in practice we find editors work nothing the worse for having a tolerable salary from a publisher, or a copious list of subscribers. The most liberal and patriotic writer never, so far as we can see, complains of finding his liberality and patriotism damped by their reward. Nay, singular as it may appear, the more he is encouraged, the more money he makes, the warmer is the complexion of his liberality. Were a new reforming Journal to be established on the broad bottom of no pay for any person concerned in its writing, publishing, or printing, we are inclined to think there would not be a very lively contest for the situations of editor, publisher, or printer. In short, look where we will—at the bar—the pulpit—the judicial bench—the ministerial office—the "gallant ship"—or the embattled field—we see that pay for services performed is admitted as a principle and acted on in practice, with the best effects. Why then should the unhappy hospital functionary be singled out as the only man who is to work for nothing—why are the feelings of human nature to be disregarded in his case—and why is he to be supposed capable of doing what nobody else is asked to do, to work well and willingly without reward?

No doubt the principle of pay may be admitted, yet exception may be taken at its mode and its amount. In the case of the hospitals, we think that the method of remuneration is by no means bad. As the charitable institutions greatly depend upon private subscriptions, and as, whether that be so or not, the funds were intended, as exclusively as possible, for charity's sake, the less taken from those funds, for purposes not strictly charitable, the better.

If a man becomes surgeon to a hospital where he is not paid, he binds himself to perform certain duties to the patients; and, if those duties are not performed, the persons who elected him have, or ought to have, the power of punishing him by suspension, or even by dismissal from his office. But pupils may be said to constitute an excrescence on a charitable institution—they are not essentially connected with it. Unless it be a surgeon's or physician's interest to pay the utmost attention to pupils, there is and there can be no efficient mode of preventing his neglect of them. The mere routine performance of specified duties is utterly inadequate to promote their advancement—there must be a disposition to direct, encourage, and assist. That disposition, we repeat, can be ensured by remuneration only. And from whom can remuneration more properly come, than from those who are to derive advantage from it?

If it were otherwise an object of importance to protect the pupil from expense, if it were better that he should pay no fees, and that his entrance into the profession should be made as cheap as possible, we might feel inclined to look for some other mode of payment than such fees afford. But, so far from it being advisable to render admission into the profession very cheap, we think it would be well if it were more difficult than it is. Our ranks are at present more than sufficiently crowded, and the rate of profit is fearfully beaten down by the ruinous struggles of starving competitors for bread. Not a town, not a hamlet, but bears evidence to this—not a medical practitioner but directly or indirectly feels it. It is an evil that has increased, is increasing, and ought to be diminished. Let the common sense of our readers decide, whether that evil will be decreased by greatly cheapening medical education. For our own parts, we do not hesitate to express our opinion, popular or unpopular, liberal or illiberal as it may appear. We think the facilities for reception in the profession should not be too great; but we do not think that, to effect the desirable purpose of preventing an indiscriminate rush into our ranks, arbitrary or unreasonable tolls should be levied. Education should not be merely made dear—it should be dear only by being extensive. Whether our numbers be great or small, our real respectability can only be secured by securing the highest amount of attainments.

We agree with our contemporary, that the offices of house-surgeon, dresser, clinical clerk, and others of the same description, should not be obtained by the payment of money. They ought to be the reward of industry and merit; at some of the metropolitan hospitals they are so. At St. George's Hospital, for example, no fee is paid for any of the situations in question. But those officers who reside in the hospital pay a small sum for their board in the institution, which is not unfair, for a young man must eat and drink, and probably it costs him less to live in the hospital than out of it.

The amount of the money pocketed by the corps d'élite is ineffably deplored. This amount must vary with the numbers of attending students. If these are many, the emolument is great—if few, it is proportionately less. As pupils select what hospital they choose, and as we cannot suppose them such fools as not to go where they think they obtain the most for their money, it is surely no crime on the part of the surgeons of a particular institution, that they receive much of this voluntary recompence. At Bartholomew's Hospital, the emoluments of the surgeons and physicians are great, because the school is in high estimation—at the Middlesex Hospital, the reputation of the neighbouring school at the London University, and the erection of its hospital, have so diminished the number of pupils, that the income of the medical officers must be small. Are Mr. Stanley and Mr. Lawrence to be censured, because they are thought such good teachers, that gentlemen insist on paying their money to them? It would be strange political economy this, to affirm that men should not be paid in proportion to their merits or their reputation. This is one of many instances of the absurdities to which an indiscriminate rage for altering and reforming leads.

We think we have disposed of the question of pay—we think we have shewn that it is just and politic to remunerate the medical officers of our hospitals, and we think we have shewn, also, that the mode of remuneration is, perhaps, the best that the case permits.

But another point remains to be considered. While we advocate the payment of medical officers, we do not advocate the principle of paying them for nothing. On the contrary, we think they ought to furnish a quid pro quo. It is imperative on them, in every point of view, both politic and moral, to return an equivalent, in the shape of assistance and instruction, for the pupil's fee. Our contemporary expends a good deal of useful indignation on this head.

"Our inquirer would then demand, with a rueful face, what sacrifice of time and labour functionaries so well paid, both directly and indirectly, bestowed upon their studies? The answer, we opine, would yet lengthen his visage, 'a very trifle, no more time or labour than is compatible with an extensive private practice; to which, indeed, they all bend their principal attention and energy. With a few solitary exceptions, they deliver no clinical lectures, by which to enlighten their juniors, the *paying* students; nor visit the bedsides of the sick oftener than once a-day, unless upon some great emergency.' We now quit our friend to make a few observations.

"Students, paying at the rate of 25 guineas each to the principal surgeon of a hospital for their instruction, are permitted to follow the heels of the latter through the different wards—from 100 to 200 at least do this at a large hospital annually. It is plain, that so great a number cannot, as things are now conducted, derive much benefit from their *soi-disant* instructors in what is called hospital practice. For how can even a fourth part of the number of students witness the conduct, or hear the opinions of the surgeon, at the patient's bedside? Ten or twelve get close to him, surround the bed, and exclude the rest from perceiving what is going on. The celerity, too, with which the business of visiting the sick in hospitals is performed, adds to the pupil's difficulties. Now, the only mode in which the surgeon could repair this error, in receiving more than he can give a fair equivalent for, in the ward-hunting business, would be by delivering, daily, clinical lectures in the theatre of the school, where there would be accommodation for all—an hour, at least, daily, should the surgeon lecture."

The picture here drawn is not strictly applicable to the present state of things. So far as the younger medical officers of hospitals are concerned, we suspect that, instead of lecturing too little, they lecture too much. The pupils are lectured to death, and the difficulty is, not to get clinical lectures delivered, but persons to attend them. An eminent man, like Sir Benjamin Brodie is attended well enough; but the junior surgeons and physicians are not attended. On this subject, we can speak with the positiveness of actual information. We repeat, that the lecturers of all sorts are so numerous, that pupils cannot be induced to attend all—a good example of the difference between theory and practice. Suppose our contemporary's recommendations adopted—suppose a daily lecture of an hour from each surgeon and physician. Generally speaking, there are four of each, and, if each is to lecture at our contemporary's pace, that would be eight clinical lectures daily. We will imagine, however, that only two clinical lectures are delivered, and that one surgeon and one physician deliver daily an hour's oration. We will suppose, too, that in addition to this, the wards of the hospital are visited at length, and with minute attention. We will suppose all this, and we venture to say that the majority of the clinical lecturers would enjoy audience of empty benches.

The fact is, that the prevalent mistake at present is the idea, that nothing is to be done but by lectures. The pupil is converted into a sort of slop-bason, into which every one empties his cup. No time is left to him for free thought or action. Yet, after all, there is nothing great effected but by an individual's own exertions. It is not what he hears, but what he sees or does. Clinical lectures are well in their way, but a diligent student, who takes his case book, and studies disease at the bed-side, will do much more than he who is lectured and crammed with other men's thoughts and experience, from morning to night. We repeat, that our pupils are over-lectured, overdosed with formulæ for ready-made knowledge. The great point is to stimulate young men to work—to set them about studying nature for themselves—to give them judicious advice and assistance—but not to spare, or attempt to spare, them the exertion and trouble of thought. The surgeon or physician should point out in his rounds, briefly and succinctly, the peculiarities of a case—he should tell the student what to watch, and what to note—should shew him the mode of investigating circumstances—and explain the reasons and mention the probable effects of treatment. A careful visit, so conducted, would be much less tiresome, and much more useful, than incessant and formal clinical lectures. If an hour's clinical lecture is given each day, it is impossible for the ordinary student to attend to that, and properly to study cases also. Practically speaking, that impossibility is openly avowed, in the lax attendance on many of the clinical lectures that are given.

Reviewers, in their arm-chairs, settle all these matters to their own satisfaction. A clinical lecture is confessedly a good thing, and, therefore, the more of it the better. These gentlemen forget that there are lectures on materia medica, on midwifery, on physic, on surgery, on botany, on medical jurisprudence, and demonstrations of, and lectures on anatomy, and dissections, and attendance at the hospital, and operations to be seen, and pathological examinations to be witnessed, all, perhaps, or most of these things, to be done on the same day. Then the student is to eat and drink, and, as he is but a human creature, it is possible that he may sometimes feel fatigue, and even occasionally some ennui; and he has books to read, and notes to copy out, and may be supposed capable of indulging a little in reflection. Enough, says the proverb, is as good as a feast —enough work is possibly better. The student, we suspect, begins to discover that it is not lectures that are now deficient, and further improvements in the system of medical education will not probably consist in the delivery of more lectures.

It is not our object to point out exactly what ought to be done, but to dispel the exaggerated notions that prevail, with respect to the supineness of the medical officers of hospitals. We are far from thinking our hospital system as perfect as it should be—there are probably sins, both of omission and of commission, in their management—but the want of lectures, we are sure, is none of them.

We do not maintain that no clinical lectures should be given. We are far from thinking that, in fact, we have been clamorous for their delivery ourselves. But we do think they should not be carried too far, and that judicious bed-side tuition is probably not carried far enough. We must recollect that pupils are young men whose stock of patience and attention is exhaustible, and that it is easier to bid them sit hour after hour at lectures, than to make them—

—— Come when we do call them.

